# State-of-the-Art Electrode Materials for Sodium-Ion Batteries

**DOI:** 10.3390/ma13163453

**Published:** 2020-08-05

**Authors:** Alain Mauger, Christian M. Julien

**Affiliations:** Institut de Minéralogie, de Physique des Matériaux et Cosmochimie (IMPMC), Sorbonne Université, UMR CNRS 7590, 4 place Jussieu, 75252 Paris, France; alain.mauger@sorbonne-universite.fr

**Keywords:** sodium, cathode, anode, sodium-ion batteries, energy storage

## Abstract

Sodium-ion batteries (SIBs) were investigated as recently as in the seventies. However, they have been overshadowed for decades, due to the success of lithium-ion batteries that demonstrated higher energy densities and longer cycle lives. Since then, the witness a re-emergence of the SIBs and renewed interest evidenced by an exponential increase of the publications devoted to them (about 9000 publications in 2019, more than 6000 in the first six months this year). This huge effort in research has led and is leading to an important and constant progress in the performance of the SIBs, which have conquered an industrial market and are now commercialized. This progress concerns all the elements of the batteries. We have already recently reviewed the salts and electrolytes, including solid electrolytes to build all-solid-state SIBs. The present review is then devoted to the electrode materials. For anodes, they include carbons, metal chalcogenide-based materials, intercalation-based and conversion reaction compounds (transition metal oxides and sulfides), intermetallic compounds serving as functional alloying elements. For cathodes, layered oxide materials, polyionic compounds, sulfates, pyrophosphates and Prussian blue analogs are reviewed. The electrode structuring is also discussed, as it impacts, importantly, the electrochemical performance. Attention is focused on the progress made in the last five years to report the state-of-the-art in the performance of the SIBs and justify the efforts of research.

## 1. Introduction

Sodium-ion batteries (SIBs) have been studied as early as the 1970s. In the 1980s, however, the lithium-ion batteries (LIBs) looked more promising [1]. Actually, material components are very similar in both cases, as the material components used as intercalation compounds can be obtained by substitution of Li and Na, like LiCoO_2_ and NaCoO_2_ [2], or other Li*M*O_2_ and Na*M*O_2_ with *M* a transition metal ion [3]. Owing to the higher energy densities obtained with LIBs, the efforts of research have soon been more focused on them, even though the research on SIBs has continued [4,5,6,7]. This can be understood if we note that Na^+^ is heavier than Li^+^ (23 g mol^−1^ against 6.9 g mol^−1^). Moreover, Na has an electrode standard potential (−2.71 V vs. SHE) less electroactive than that of Li (−3.04 V vs. SHE), which results in lower cell voltages for sodium materials. The combined effect of heavier cation weight and lower cell voltage inevitably implies that SIBs have a smaller energy density than LIBs [8]. Other advantages of LIBs include longer cycle life, lower reactivity, less moisture/nitrogen sensitivity.

Nevertheless, an important renewed interest in SIBs has been observed in the recent years [9]. This renewed interest is evident from the effort in research on SIBs, which has been reviewed in [10,11,12] for anodes and [13,14,15,16] for cathode materials. Different reasons justify this renewed interest. First, the similarity of the methodologies, materials and processing make SIBs a “drop-in” technology for LIBs [17,18]. Another advantage of SIBs is the lower cost. Typically, a quarter of Li reserves are expected to be depleted just for electric vehicle (EV) applications by 2050 [19,20]. This may not be meaningful since more reserves will presumably be found in forthcoming years as we will look for them. However, the reserves of lithium will always be smaller than those of sodium, which is not only abundant in the earth’s crust, but also can be extracted from the salt of the oceans. One consequence is a big difference in price between lithium and sodium metals. For comparison, the price of the carbonates that constitute a brine source of Li and Na, are US$ 6600 per Mt for Li_2_CO_3_ and US$ 60 per Mt for Na_2_CO_3_ at the London Metal Exchange. This was taken as an argument to claim that the cost of SIBs will be much smaller than that of LIBs. This argument, however, is misleading, because of the huge drop in the price of LIBs. In 2018, the average price of la lithium-ion battery was 127 $/kWh at the cell level, 176 $/kWh at the pack level [21]. This is only an average, and in particular, the Tesla’s batteries cost in 2019 reduced to 127 $/kWh [22]. Peters et al. published recently the first detailed economic assessment of 18650-type SIB cells with a layered oxide cathode and a hard carbon anode, based on existing data-sheets for pre-commercial battery cells, and compared the results with those of LIB cells with lithium-nickel-manganese-cobalt-oxide cathodes (NMC) and with lithium-iron-phosphate cathodes (LFP) [23]. The cell costs were calculated independent of their final application, for a plant throughput of 200 million battery cells per year. For the SIB, a cell price of 223 $/kWh is obtained, compared to 229 $/kWh for the LFP and 168 $/kWh for the NMC 18650-type cells. The intuitive idea that the price of SIBs would be lower than that of LIBs simply because lithium is more expensive than sodium is thus wrong. The reason is the important impact of the different storage capacities on the cost, due to the different energy densities provided by these different chemistries. This is the direct consequence of the weight penalty, as the Na^+^ ion is heavier than that of Li^+^. For some applications like electric vehicles, the SIBs will not be competitive with LIBs, because the energy density that determines the range of the cars is considered as the main parameter in the business plan of the manufacturers (although we have drawn attention to the safety concerns here [24]). The consideration of both the energy density and price also explains why the NMC has replaced LFP in the EVs. Another limitation comes from the more sluggish kinetics observed in SIBs, coming from the fact that the Na^+^ ions are not only heavier but also bigger than Li^+^ ions: the ionic radius of Li^+^ is 0.76 Å, while that of Na^+^ is 1.02 Å. Consequently, the rate capability of SIBs is also smaller than that of LIBs. Despite the performance of NMC, LFP is widely used for stationary applications because it is it much safer, with a longer cycle life [25]. Since the LFP-LIB is more expensive than the SIB, we can conclude that the SIB will not substitute to NMC for automotive applications, but is a promising substitute to LFP for stationary applications if SIBs can reach the same level of safety and cycle life. That is the reason for the numerous works devoted not only to the electrodes for SIBs, reviewed in [17,26,27], but also for their electrolytes either liquids [28] or solids [29,30] and their salts [31]. Another recent review is devoted to electrolyte/electrode interfaces (SEI) in SIBs [32]. Other reviews focus attention on Na-ion batteries with high power density [33,34]. Indeed, the Na-ion batteries have reached the stage of commercialization by two companies. Faradion Ltd. is a UK-based startup company established in 2011, using Na_a_Ni_1-x-y-z_Mn_x_Mg_y_Ti_z_O_2_ as the cathode material and hard carbon anode. It demonstrated the first generation of its Na-ion chemistry in an E-bike platform using a 400-Wh battery pack. The other company is Novasis Energies, Inc. using Prussian blue analog as the cathode and hard carbon anode. The areal loadings of their electrodes are above 2 mA·h·cm^−2^. The cell energy density is 100–130 Wh·kg^−1^ or 150–210 Wh·L^−1^. The electrochemical performance of active materials and full cell performance of the batteries developed by these two startup companies can be found in [35], and illustrates the readiness level of SIB technology [36].

Organic materials are considered as emerging candidates as an alternative to inorganic-based materials, because they are cheaper, safe, and they are environmentally friendly, with high theoretical capacity [37,38]. However, they still suffer for several drawbacks that postpone any practical application. Since we have recently devoted an exhaustive review on the state-of-the-art on the organic-based materials for lithium and sodium batteries, we simply guide the readers to it [39]. Therefore, the present review is focused on the recent progress that has been achieved in inorganic electrode materials.

## 2. Cathode Materials

**Figure 1:**
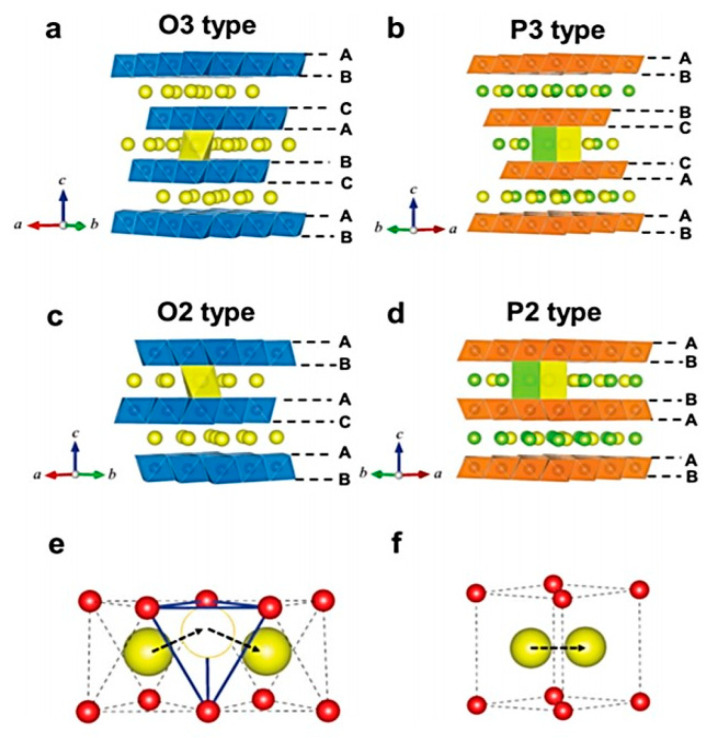
Schematic illustrations of definitions of layered NaTMO_2_ phases of (**a**) O3 type, (**b**) P3 type, (**c**) O2 type, and (**d**) P2 type. Illustrations of Na^+^ ion diffusion pathways through (**e**) indirect tetrahedral sites in the O-type stacking sequence and (**f**) direct prismatic sites in the P-type stacking sequence. Reproduced with permission from [41] Copyright 2016 Royal Society of Chemistry.

Na-based layered oxide materials are a viable Na-ion battery cathode [40]. Their theoretical capacity is high (up to 244 mA·h·g^−1^ for O3-NaMnO_2_) and the synthesis process is rather simple. They are classified O3-, P2-, and P3-types depending on the stacking sequence of oxygen layers (O3: *ABCABC* stacking; P2: *ABBA* stacking; P3: *ABBCCA* stacking) (see Figure 1) [41]. A review on the structure–function–property relationship of layered oxides for SIBs has been published by Wang et al. [42].

### 2.1. P2-Layered Oxide Materials

To stabilize the P2-sructure, the concentration of Na ions must be reduced and attention in recent years has been focused on Na*xM*O_2_ (*M* stands for a transition metal ion) with *x* = 2/3 (the P2 phase cannot be maintained at larger *x*). P2-Na_x_CoO_2_ is known for its poor cycle ability, but recent progress has been achieved by controlling the morphology. Na_0.7_CoO_2_ spheres 5 μm in diameter demonstrated a capacity of 125 mA·h·g^−1^ at 0.04C (about 5 mA·g^−1^) in the voltage range 2.0–3.8 V vs. Na^+^/Na, and the capacity retention at 0.4 C was 86% after 300 cycles [43]. Na_0.7_CoO_2_ nanosheets orderly grown on Ni foam delivered an areal capacity of 1.16 mA·h·cm^−2^ at 1C rate, with a prolonged cycling life. More than 50 mA·h·g^−1^ capacity was preserved after 1100 cycles at 6C, and even at a larger rate of 15C, a capacity of 57.8 mA·h·g^−1^ maintained. [44]. Among P2-Na*xM*O_2_, a partial substitution of the cation has been used in recent works to stabilize the P2-structure (and thus increase the capacity retention). This effect has been evidenced in Na_0.7_CoO_2_, where the Mg substitution also increases the rate capability owing to an increase of the Na^+^ diffusion coefficient [45]. In addition, the cationic substitution improves the cycling life due to the suppression of the Na^+^/vacancy ordering condition, experienced in P2-Na_x_Co_1-y_(Mn_2/3_Ni_1/3_)_y_O_2_ [46], and also Ca-substitution [45,47]. This is also true with Ni substitution, but only below a solubility limit [48], and Ti substitution, but only for Ti concentration smaller than 10% [49]. Nevertheless, the sodium ion battery based on P2-Na_x_CoO_2_ cathode still has an energy density that is too small for practical applications [49,50].

The substitution effect is even more important in the case *M* = Mn because the substitution is mandatory to suppress the Jahn–Teller (JT) distortion due to Mn^3+^ ions. As a result, P2–Na_0.67_Ni_0.25_Mg_0.1_Mn_0.65_O_2_ delivers a capacity of 100 mA·h·g^−1^ at C/10 rate with a retention of 87% after 100 cycles in the voltage region of 1.5–4.2 V [51]. Note that the substitution of Ni alone for Mn does not give good results because of the P2–O2 transition at low voltage (below 2.3 V) which damages the structural stability and thus the capacity retention. One solution to avoid this effect is the limitation of the operating voltage range to 2.3–4.1 V [52,53], but this is detrimental to the capacity and thus the energy density. The other solution is the additional substitution of Mg [51] or a transition element, among them Ti [54] but the capacity retention was tested at 0.1C on 20 cycles only, or over 50 cycles at very slow rate (C/50) [55]. The role of the Mg substitution in P2-Na_2/3_Ni_1/3-x_Mg_x_Mn_2/3_O_2_ (0 ≤ *x* ≤ 0.2) has been investigated by Tapia-Ruiz et al. [56]. In particular, they showed that the Mg substitution increases the diffusion coefficient of Na. In P2-Na_0.67_Mn_x_Co_1-x_O_2_, Co-rich phases exhibit a high structural stability and superior capacity retention, whereas Mn-rich phases discharge higher capacities [57].

Improved results were obtained with Fe [58]. The Na_0.5_[Ni_0.23_Fe_0.13_Mn_0.63_]O_2_ cathode delivered 200 and 150 mA·h·g^−1^ at 15 mA·g^−1^ (C/10) and 100 mA·g^−1^, respectively. At current density 100 mA·g^−1^, the capacity was retained at 125 mA·h·g^−1^ after 100 cycles [59]. Almost the same initial capacities were obtained in P2-Na_2/3_[Fe_0.5_Mn_0.5_]O_2_ but with a much poorer cycle ability, due to a structural phase transition [60] and the fact that the migration of Fe^3+^ into tetrahedral sites in the interlayer space is avoided by the Ni substitution [61]. P2-Na_2/3_[Fe_1/4_Co_1/4_Mn_0.5_]O_2_ exhibited a high rate performance of 130 mA·h·g^−1^ at 30C [62], but the cycle life was poor, due to a P2–O2 transition was accompanied by a large lattice volumetric contraction at 4.2 V [63]. The sol-gel synthesis of this material improved the cycle ability, with a first discharge capacity of 157 mA·h·g^−1^ and a capacity retention of 91 mA·h·g^−1^ after 100 cycles at 130 mA·g^−1^ [64].

The substitution of other elements that are different from transition metals, namely Li, Cu, was also investigated. In particular, P2-Na_0.85_Li_0.17_Ni_0.21_Mn_0.64_O_2_ delivers a capacity of 95–100 mA·h·g^−1^ between 2.0 and 4.2 V with a capacity retention of 98% over 50 cycles at C/10 [65]. The Li^+^ ions remain fixed in the transition layer [65,66], allowing more Na ions to reside in the prismatic sites at high voltage, the reason why the P2-structure is stabilized [67]. The Cu substitution improves the capacity retention with respect to Mg or Ni doping, and also improves the rate capability [68]. Even at a current rate of 1000 mA·g^−1^, the capacity retention of Na_0.67_Cu_x_Mn_1-x_O_2_ is raised to 76.6% after 500 cycles for *x* = 0.33 [69]. Indeed, an important progress has been made recently by divalent Zn-doping of P2-type Mn-based cathodes, since Zn-doping reduces the amount of the JT distorted Mn^3+^ centers, and thus improves the structural stability. For instance, Zn-doped Na_0.833_[Li_0.25_Mn_0.75_]O_2_ (NLMO) delivered a capacity 162  mA·h·g^−1^ very stable over 200 cycles at 0.2C [70]. This important improvement with respect to the electrochemical properties of the undoped samples has been attributed to the localization of Zn^2+^ in the Na-layer, which stabilizes the diffusion channels during charge/discharge processes.

**Figure 2:**
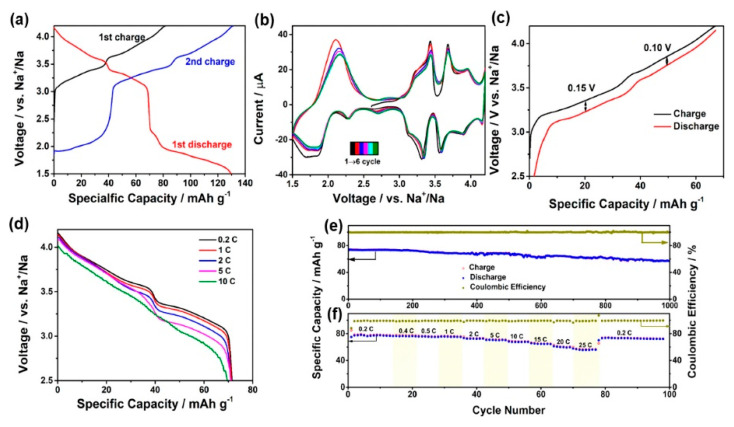
(**a**) Charge and discharge profiles of P2-layered Na_0.7_[Mn_0.6_Ni_0.4-x_Mg_x_]O_2_ with *x* = 0.02 (MNM-2) between 1.5 and 4.2 V. (**b**) CV curves of MNM-2 between 1.5 and 4.2 V (scan rate: 0.1 mV#xB7;s^−1^). (**c**) Charge and discharge profiles of MNM-2 in the second cycle between 2.5 and 4.2 V. (**d**) Charge and discharge profiles of MNM-2 at different current densities from 0.2 to 10C. (**e**) Cycle performance at 1C for 1000 cycles. (**f**) Rate capability of MNM-2 from 0.2 to 25C between 2.5 and 4.2 V. Reproduced with permission from [73]. Copyright 2019 The American Chemical Society.

Mg-doped Na[Li_0.25_Mn_0.75_]O_2_ has the same Mn electronic structure as Zn-doped Na[Li_0.25_Mn_0.75_]O_2_ and thus experiences the same reduction of the JT distortion. Indeed, Na_2/3_Mn_1-y_Mg_y_O_2_ (*y* = 0.05 and 0.1) has an improved structural stability with respect to the undoped material [71]. For the same reason the Mg-doping suppressed the P2-O2 phase transition in Na_0.67_Mn_0.67_Ni_0.33_O_2_ [72]. Another example of the stabilization of the P2 structure by the Na-site Mg substitution was demonstrated with the superior electrochemical performance of Na_0.7_Mg_0.05_[Mn_0.6_Ni_0.2_Mg_0.15_]O_2_ [73] (see Figure 2). Due to Na^+^/vacancy-order superstructures, undoped P2-layered oxides suffer from multiple voltage plateaus. Such is the case of pristine Na[Li_0.25_Mn_0.75_]O_2_ charge–discharge profile. On the other hand, the profile of the Mg-doped material in Figure 2a shows that Mg-doping was effective to smooth the charge–discharge profile, which is beneficial for the capacity retention. After first charging, the six redox peak couples (see Figure 2b) are well overlapped, indicating the high reversibility. The charge/discharge profiles in the second cycle within a narrow voltage window between 2.5 and 4.2 V are reported in Figure 2c. The polarization of the plateau at higher voltage is 0.10 V, smaller than that of the other plateau, demonstrating that the Na-poor phase has enhanced kinetics for Na-ion and electron transportation. When cycled between 2.5 and 4.2 V at 1C rate, this cathode demonstrated a capacity of 70 mA·h·g^−1^, with 79% capacity retention after 1000 cycles. Na_2/3_Ni_1/6_Mn_2/3_Cu_1/9_Mg_1/18_O_2_ cathode material consisting of multiple-layer oriented stacking nanoflakes was proposed by Xiao et al. [74]. This cathode material demonstrated a good rate capability (64.0 mA·h·g^−1^ and 11.4 kW·kg^−1^ at 30C).

Wang et al. noticed that smaller Na^+^ diffusion coefficient is observed in P2-type layered oxides exhibiting Na^+^/vacancy-ordered superstructures because of strong Na^+^-Na^+^ interaction in the alkali metal layer and charge ordering in the transition metal layer [75]. They showed that such Na vacancy ordering can be avoided by choosing the transition metal ions with similar ionic radii and different redox potentials like Cr^3+^ and Ti^4+^. The full symmetric cell with P2-Na_0.6_[Cr_0.6_Ti_0.4_]O_2_ as both positive and negative electrodes in the NaPF_6_-based electrolyte delivered a capacity of 80 mA·h·g^−1^ at 1C (76 mA·g^−1^), retained at 65 mA·h·g^−1^ after 100 cycles. The capacity at 12C was still 75% of the capacity at 1C.

In the same spirit, an increase of the diffusion coefficient can also be obtained by mixing two structures. In particular, P2-O3 composites were found efficient to improve the rate capability owing to a better sodium diffusion. Among them, Na_0.66_Li_0.18_Mn_0.71_Ni_0.21_Co_0.08_O_2+d_ delivered a capacity of 134 mA·h·g^−1^ at 1C [76]. Another example is the P2-P3 composite Na_0.66_Co_0.5_Mn_0.5_O_2_, which demonstrated much better electrochemical properties than the P2-sample with the same composition. The composite delivered an initial discharge capacity of 86.5 mA·h·g^−1^ maintained at 78.9 mA·h·g^−1^ at the 100th cycle at 10C [77].

Another strategy to stabilize the P2 phase was the coating of the P2-particles with a protective layer. Without coating, the P2-O2 crystal phase transition and the large volume change of the O2 phase (more than 20%) is difficult to avoid, since the O2 structure has a lower formation energy density than the P2 structure at high voltage. The coating aims to mitigate the volume change during cycling, and suppress the side reaction during long cycling within the high voltage window. This strategy has been used in particular with P2-Na_2/3_[Ni_1/3_Mn_2/3_]O_2_, which is a high-voltage cathode material for Na-ion batteries with a theoretical capacity of 173 mA·h·g^−1^ and a long operation voltage plateau of 4.2 V. Therefore, this cathode is attractive, but has a very poor cycle ability. A remarkable improvement was achieved by coating the particles with Al_2_O_3_ [78]. This coating was able to suppress unfavorable side reactions with the electrolyte at high voltage, as well as exfoliation of the metal oxide layers, leading to 73.2% capacity retention over 300 cycles. During cycling, the coating formed polymeric species such as poly(ethylene oxide), which provide flexibility in the SEI, and this suppressed exfoliation of the P2 layered material [79]. More recently, Na_2/3_[Ni_1/3_Mn_2/3_]O_2_ was modified with an ionic conducting NaPO_3_ coating layer via melt-impregnation [80]. The corresponding full cell with a hard carbon anode demonstrated a capacity of 135 mA·g^−1^ after 300 cycles at 40 mA·g^−1^ (0.2C), which corresponds to 73% retention.

As an alternative to the coating of Na_2/3_[Ni_1/3_Mn_2/3_]O_2_, rational designing of the transition-metal layer can be used to achieve both water-stable and Na^+^ vacancy disordering structures. Partial substitution of the transition metal ions with Fe or Co suppresses the Na^+^ vacancy ordering arrangement, but makes this material vulnerable to water molecules. Density functional theory calculations reveal that the water-stability of the layered oxide cathode can be correlated to the surface adsorption energy of H_2_O molecules [81]. The Co/Mn and Fe/Mn units exhibit a much lower adsorption energy than that of the Li/Mn unit. Therefore, Zhang et al. introduced Li to obtain the water-stable Na_2/3_Li_1/9_Ni_5/18_Mn_2/3_O_2_ cathode. This cathode exhibited exhibit high rate capability with high retention of 78% (72 mA·h·g^−1^) at 20C, and excellent cycling stability (87% capacity retention after 1000 cycles) [81]. Moreover, even after water-soaking treatment for 24 h, this cathode maintained its original crystal structure as well as electrochemical performance while the Na_2/3_Fe_1/9_Ni_1/6_Mn_2/3_O_2_ and Na_2/3_Co_1/9_Ni_1/6_Mn_2/3_O_2_ cathodes were easily invaded by H_2_O molecules and exhibited poor electrochemical properties. Therefore, the partial substitution of Li^+^ for Ni^2+^ had two positive effects: First, it eliminated the Na^+^ vacancy ordering in the Na layer, leading to a solid solution behavior with superior Na^+^ kinetics during the insertion/extraction processes. Second, this strategy was effective for the achievement of a water-stable cathode with high rate capability for SIBs.

Another strategy to increase the structural stability during cycling consists in engaging crystal water in the interlayer space of sodium manganese oxide under the birnessite framework [82]. The crystal water enhances Na ion diffusion both in the crystal host and at the interface. The water co-deintercalation was found efficient in mitigating the interlayer expansion during the high potential charging. In particular, after reacting with water, α-NaMnO_2_ translates into crystal water containing Na-birnessite with a large interlayer distance of 7.15 Å, which results in an improvement of the cycling life [83]. More recently, Shan et al. demonstrated this process in Na_0.27_MnO_2_∙0.63H_2_O which is crystallized in the birnessite (δ-MnO_2_) phase, using in-situ XRD experiments [84]. In this material, a co-deintercalation of water molecules along with Na-ion at the high potential charging could stabilize the layered structure from over-expansion of the interlayer distance. The co-deintercalation of water molecules along with Na-ions resulted in a shrinkage of the interlayer distance, and thus stabilizes the layered structure against further expansion of the interlayer distance at higher voltages while sustaining an intensive redox process. Disordered Na_0.27_MnO_2_ structure (defined as a material also crystallized in the birnessite, but with smaller coherence length than ordered Na_0.27_MnO_2_) is also an advantage, since it allows continuous and smooth structural evolution during the charging and discharging processes without phase transitions and possesses highly exposed (001) planes with low density of active edge sites. As a result, this sodium-rich disordered birnessite for aqueous sodium-ion electrochemical storage demonstrated a much-enhanced capacity and cycling life (83 mA·h·g^−1^ after 5000 cycles in full-cell).

### 2.2. O3-Layered Oxide Materials

**Figure 3:**
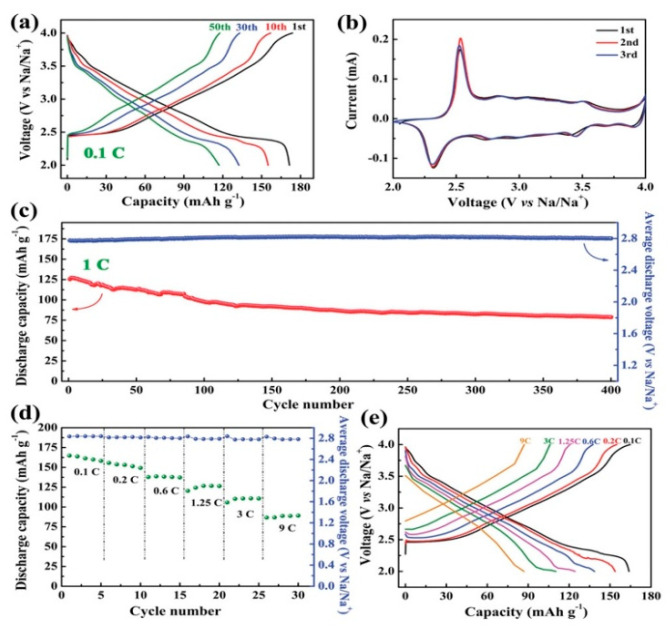
O3-LiNi_0.82_Co_0.12_Mn_0.06_O_2_ cathode: (**a**) charge/discharge curves at 0.1C, (**b**) cyclic voltammogram curves at a scan rate of 0.05 mV s^−1^, (**c**) long-term cycle life and average discharge voltage at 1C, (**d**) discharge capacity and average discharge voltage, and (**e**) charge/discharge curves at different rates. Reproduced with permission from [90]. Copyright 2019 Wiley.

O3-layered materials used as cathodes are able to deliver a higher discharge capacity. However, the migration of Na ions from one prismatic site to another requires only one transit of the rectangular face. Therefore, the P2 phase is beneficial for being used as a high rate cathode. Carbon-coated NaCrO_2_ delivers a capacity of 120 mA·h·g^−1^ at a current density of 20 mA·g^−1^, with a good capacity retention over 50 cycles [85]. Moreover, the capacity was maintained at 99 mA·h·g^−1^ at 150C, demonstrating an excellent rate capability. Like in the case of P2-type materials, the mixing of transition metal elements was found efficient to stabilize the O3 structure during cycling. The incorporation of Ni into the Fe/Mn transition metal layer was explored in [86]. O3-NaFe_0.2_Mn_0.4_Ni_0.4_O_2_ undergoes a phase transition from the P3 to P2 phase, which has a smaller interslab distance than the P3′′ phase in the high-voltage region, resulting in stable cycle performance. The incorporation of Cu into the Fe/Mn transition metal layer not only enhances the stability but also improves the reversibility and kinetics [87]. The O3-Na_0.9_[Cu_0.22_Fe_0.30_Mn_0.48_]O_2_ electrode cycled in a voltage range of 2.5–4.05 V versus Na^+^/Na at a current rate of 0.1C (10 mA·g^−1^) in a half-cell delivered a capacity of 100 mA·h·g^−1^ based on the mass of the cathode. This capacity was found to be very stable over 100 cycles. The full cell O3-Na_0.9_[Cu_0.22_Fe_0.30_Mn_0.48_]O_2_//hard carbon with NaPF_6_-based electrolyte cycled in the voltage range 1–4.05 V delivered a capacity of 300 mA·h·g^−1^ based on the mass of the anode. X-ray absorption near-edge spectroscopy (XANES) spectra showed that copper and iron are both electrochemically active and the redox couples of Cu^2+^/Cu^3+^ and Fe^3+^/Fe^4+^ are mainly responsible for the charge compensation during the electrochemical process. Core-shell like structured particles with a composition gradient from the inner-end Na[N_0.75_Co_0.02_Mn_0.23_]O_2_ to the outer-end Na[Ni_0.58_Co_0.06_Mn_0.36_]O_2_ was proposed by Hwang et al. [88]. As a result, a discharge capacity of 157 mA·h·g^−1^ at a current rate of 15 mA·g^−1^ was obtained (per mass of oxide), with a capacity retention of 80% (125  mA·h·g^−1^) during 300 cycles in combination with a hard carbon anode, and a rate capability of 132.6  mA·h·g^−1^ (1.5 A·g^−1^, 10C rate). This high performance was attributed to the reaction based on Ni^2+/3+/4+^ and the beneficial effect of the radially aligned hierarchical columnar structure on the protection against corrosion of the electrolyte. Higher capacities can be achieved by Li-doping. In particular, Na_0.78_Li_1.167_Ni_0.25_Mn_0.583_O_2_ tested between 1.5 and 4.2 V with a current density at 125 mA·g^−1^ delivered a capacity of 190 mA·h·g^−1^ after 30 cycles, and 160 mA·h·g^−1^ at 1.25 A·g^−1^ current density [89]. The full cell with SnS_2_/rGO as an anode delivered an initial capacity of ∼210 mA·h·g^−1^ (capacity based on cathode weight) when cycled in the range 1.5–4.5 V, 165 mA·h·g^−1^ after 50 cycles. Therefore, the initial capacity is larger than the result obtained with the hierarchical columnar structure mentioned above, but the capacity retention is smaller. O3-type Na_0.75_Ni_0.82_Co_0.12_Mn_0.06_O_2_ delivered a reversible capacity of 171 mA·h·g^−1^, a stable discharge voltage of 2.8 V, and maintained a discharge capacity of 80 mA·h·g^−1^ with 65% capacity retention after 400 cycles at 1C (Figure 3) [90]. Here, the electrochemically active species is Ni, while Co only participates in the charge compensation at high voltage, and Mn plays a role in stabilizing the structure during Na^+^ extraction/insertion. Indeed, the charge/discharge curves in Figure 3a are very smooth, thus avoiding the voltage plateaus associated to the gliding of transition metal oxide slabs and Na^+^/vacancy ordering. A pair of sharp redox peaks at 2.5 V (oxidation) and 2.3 V (reduction) in Figure 3b reveals the phase transition between O3 and P3. The highly coincident curves in the initial three cycles for Na-NCM811 indicate a good reversibility of phase transition.

Other Na-deficient O3-type cathodes were investigated but did not compete with the results reported above. In particular, a capacity of 85 mA·h·g^−1^ at 2.8 V vs. Na^+^/Na with a redox reaction of Ni^4+^/Ni^2+^ and Ti^4+^/Ti^3+^ was delivered by O3-Na_0.8_Ni_0.4_Ti_0.6_O_2_ [91]. A detailed analysis of the structural evolution of O3-Na_0.67_Fe_0.67_Mn_0.33_O_2_ can be found in [92].

We have already mentioned the role of Mg^2+^ for Mn^3+^ in improving the electrochemical properties of P2-layer compounds by reduction of the JT distortion. This is also true for O3-structured materials. Enhanced properties of O3-NaMn_0.48_Ni_0.2_Fe_0.3_Mg_0.02_O_2_ under the effect of the Mg-doping has been investigated by Zhang et al. [93]. This Mg-doped material used as an electrode cycled in the range 1.5–4.2 V delivered a capacity of 136 mA·h·g^−1^, with a capacity retention of 99% after 100 cycles, while the capacity was 117 mA·h·g^−1^, with a capacity retention of 81% after 100 cycles for the undoped material.

Reviewing the structure–function property for the iron- and manganese-based compounds, Chen et al. [94] concluded that the O3-type iron- and manganese-containing NaNi_1/3_Fe_1/3_Mn_1/3_O_2_ and Na_0.9_Cu_0.22_Fe_0.30_Mn_0.48_O_2_ oxides demonstrate the most realistic commercial perspectives for SIBs, based on good air stability, high first cycle coulombic efficiency, electrochemical performance, and low manufacturing expenditure. First, the Fe^3+^/Fe^4+^ redox couple at 3.5 V is the highest among all the redox couples of transition metals for Na-ion batteries, while Mn is efficient to suppress the irreversible phase transition of trivalent iron ions migrating to the vacant adjacent tetrahedral sites.

### 2.3. Other Oxides

The orthorhombic compound Na_0.44_MnO_2_ (space group *Pbam*) presents large tunnels allowing this cathode material to reach high rate capability, particularly under the form of nano-wires or nano-rods [95,96,97,98,99]. However, the cycle ability is too small, due to the JT distortion associate to Mn^3+^. To avoid this effect, partial substitution of Ti and Fe for Mn has been investigated [95,96]. The best result was obtained with Na_0.61_[Mn_0.61-x_Fe_x_Ti_0.39_]O_2_ delivering a capacity of 90 mA·h·g^−1^ [97,98]. This capacity is small, but partly compensated by the fact that the average operating potential is high (3.56 V vs. Na^+^/Na).

Avoiding the problem of the JT distortion with the manganese, vanadium oxide-based cathode materials have much better cycle ability [99]. A graphene-coated VO_2_ electrode retained a capacity above 110 mA·h·g^−1^ rafter 1500 cycles at a current density of 18 A·g^−1^ [100]. This is just an example of the interest in the use of graphene to make a composite metal oxide@graphene as an electrode (cathode or anode) for SIBs [101,102] Nanowire-interconnected V_2_O_5_∙nH_2_O delivered a high capacity of 338 mA·h·g^−1^ at a low C-rate of 0.05 A·g^−1^, but the rate capacity in absence of the highly conductive graphene was much smaller as the capacity was reduced to 96 mA·h·g^−1^ at 1.0 A·g^−1^ [103]. A composite electrode synthesized through growing V_2_O_5_ nanosheet array on free-standing hard carbon fiber fabric by solvothermal reaction delivered a capacity of 241 mA·h·g^−1^ at 50 mA·g^−1^ and 77 mA·h·g^−1^ at 1 A·g^−1^. The capacity maintained at 184 mA·h·g^−1^ after 100 cycles at 100 mA·g^−1^ [104].

### 2.4. Polyanionic Compounds

The success of LiFePO_4_ olivine as a cathode material for Li-ion batteries was the motivation of many works on Na_x_FePO_4_, but the results were disappointing, except for NaFePO_4_ in the maricite phase that becomes electroactive when prepared as nano-sized particles [105]. As a cathode, it delivered an initial capacity of 142 mA·h·g^−1^ with 95% retention after 200 cycles at low rate. This was the motivation for the search of polyanionic compounds.

**Figure 4:**
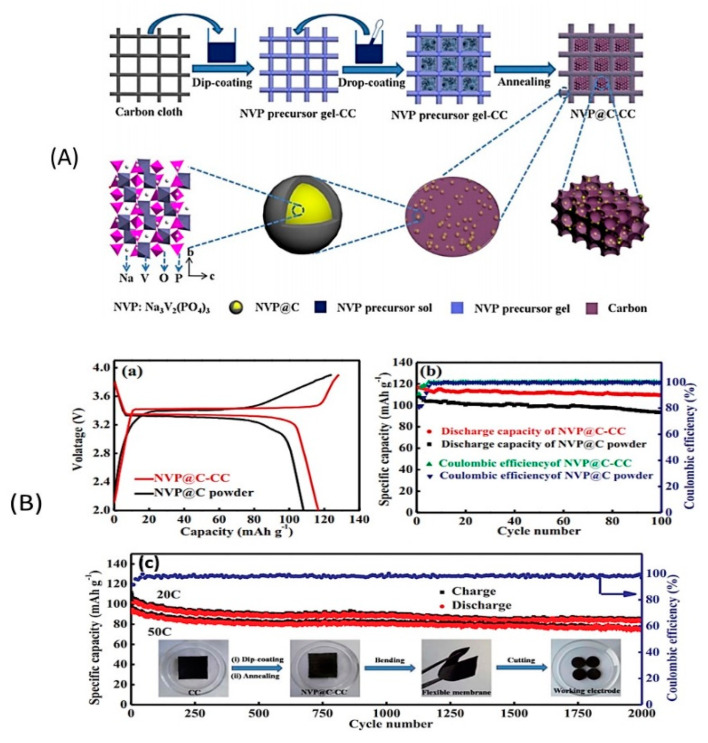
(**A**) Illustration of the preparation process of binder-free NVP@C-CC membrane, which includes the loading of Na_3_V_2_(PO_4_)_3_ (NVP) precursor on carbon cloth (CC) by dip-coating and drop-coating, followed by an annealing treatment in N_2_ atmosphere. In NTP@C, the C content was 3.2 wt.%. The mass loading of NVP@C in NVP@C-CC membrane was calculated to be 20% (3.5 mg·cm^−2^). (**B**) Electrochemical properties of this membrane. (**a**) Voltage profiles of NVP@C-CC and NVP@C powder in the voltage range from 2 to 3.9 V vs. Na^+^/Na at 1C rate. (**b**) Cycling performance and corresponding Coulombic efficiency of NVP@C-CC and NVP@C powder at 1C rate. (**c**) The long-term cycling stability and corresponding Coulombic efficiency of NVP@C-CC at 20 and 50C. The inset in (**c**) is digital photographs of the as-prepared bind-free NVP@C-CC membrane and the flexible membrane can be cut into the disks of ≈ 1.54 cm^2^ directly as the working electrode. Reproduced with permission from [109]. Copyright 2018 Elsevier.

Sodium superionic conductor (NASICON) Na_3_V_2_(PO_4_)_3_ was recognized as a promising cathode material since a long time, but its electrochemical performance has been enhanced these last years by the fabrication of nano-composites with carbon [106]. In particular, hierarchical carbon framework wrapped Na_3_V_2_(PO_4_)_3_ delivered a capacity of 115 mA·h·g^−1^ at 0.2C with outstanding cycle ability (54% capacity retention after 20,000 cycles), and high rate capability (38 mA·h·g^−1^ at 500 C). [107]. Carbon-coated Na_3_V_2_(PO_4_)_3_/C in a porous graphene network as a cathode exhibited a very high rate capability, delivering a capacity of 86 mA·h·g^−1^ at 100C with 64% retention after 10,000 cycles [108], owing to the combination of the high ionic conductivity of NASICON and the high electrical conductivity of the graphene network. More recently, carbon-coated Na_3_V_2_(PO_4_)_3_ uniformly anchored on the fibers of a carbon cloth was used as a cathode with high mass loading of 20 wt.% (3.5 mg·cm^−2^) (Figure 4) [109]. This anode delivered 82.0% capacity retention over 2000 cycles at 20C, and demonstrated a high rate capacity (96.8 mA·h·g^−1^ at 100C and 69.9 mA·h·g^−1^ at 200C). This illustrates the higher performance of binder-free and self-supporting electrode. Other examples will be reported along this review, and will be discussed later on. A 3D porous skeleton–supported Na_3_V_2_(PO_4_)_3_/carbon composite demonstrated high-rate capability (78 mA·h·g^−1^ at 192C, approaching 76.9% of the initial capability of 98.6 mA·h·g^−1^ at 0.5C), remarkable cycling stability (98.4% retention after 800 cycles at 1C, 91.4% retention after 2000 cycles at 10C), and outstanding high-rate endurance (76.0% capacity retentions after 3000 cycles at 100C) [110].

Chen et al. introduced another NASICON-type cathode element, Na_3_V_2_(PO_4_)_3_N, and used an N-doped graphene oxide-wrapped Na_3_V_2_(PO_4_)_3_N composite with a uniform carbon coating layer as a 4 V class cathode for SIBs [111]. This cathode delivered specific capacities of 78.9 mA·h·g^−1^ at 0.1C (1C = 80 mA·g^−1^) and 59.2 mA·h·g^−1^ at 30C. The capacity retention of 91.0 % and 75.9 % could be achieved at 1C (800 cycles) and 10C rate (5000 cycles), respectively.

Gao et al. used a sol-gel synthesis to obtain 200 nm particles of Na_3_MnZr(PO_4_)_3_ coated in situ with a thin carbon layer. Na_3_MnZr(PO_4_)_3_ crystallizes in the rhombohedral NASICON structure. Used as a cathode, it delivered a capacity of 105 mA·h·g^−1^ at 0.1C rate, and a capacity retention of 91% was demonstrated at 0.5C after 500 cycles [112]. Therefore, the material was stable enough and the particles small enough to avoid any problem related to the JT distortion associated with Mn^3+^. The synthesis process here was important, since prior works found poor electrochemical results with samples prepared by solid-state reaction, which led to bigger particles, and possibly non uniform carbon-coatings.

Na_3_MnTi(PO_4_)_3_/C hollow microspheres with an open and stable NASICON framework were synthesized by a spray-drying-assisted process [113]. As an anode, this composite demonstrated a capacity of 160 mA·h·g^−1^ at 0.2C. When cycled at 2C, the capacity was capacity of 119 mA·h·g^−1^ with ≈ 92 % capacity retention after 500 cycles.

**Figure 5:**
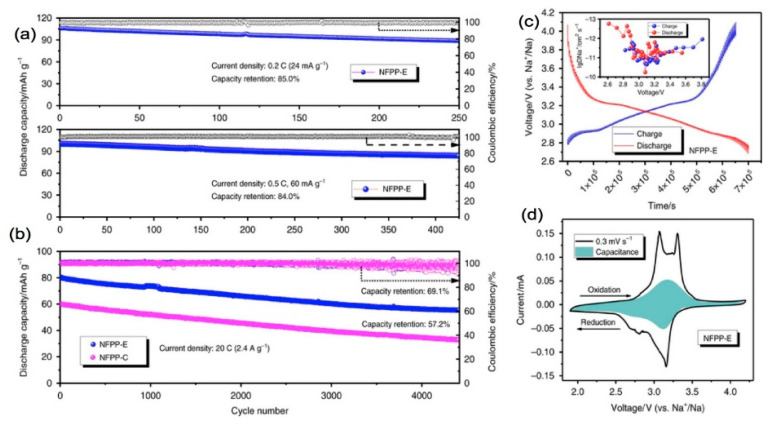
Performance of nanosized Na_4_Fe_3_(PO_4_)_2_(P_2_O_7_) plates (NFPP-E) and microporous Na_4_Fe_3_(PO_4_)_2_(P_2_O_7_) particles (NFPP-C) prepared by sol-gel. (**a**) Cycling stability of NFPP-E electrodes over 250 cycles at 0.2C and 430 cycles at 0.5C. (**b**) Long-term cycling stability (4400 cycles) at high rate (20C) for both NFPP-E and NFPP-C electrodes. (**c**) Galvanostatic intermittent titration technique (GITT) curves of NFPP-E material for both charge and discharge processes. The inset is the chemical diffusion coefficient of Na^+^ ions as a function of voltage calculated from the GITT profile (after 30 cycles, current density: 0.05C). (**d**) The calculated capacitance contribution (shadowed area) to the CV curve of NFPP-E at the scan rate of 0.3 mV·s^−1^. Reproduced with permission from [116]. Copyright 2019 Springer Nature.

Na_4_Fe_3_(PO_4_)_2_(P_2_O_7_), with its mixed crystalline framework represented by the ortho-pyrophosphates, possesses 3D sodium diffusion pathways in the study crystal framework of a typical NASICON-type structure and is thus a good candidate as a cathode for SIBs [114]. Carbon-coated nanosized Na_4_Fe_3_(PO_4_)_2_(P_2_O_7_), with its mixed crystalline framework represented by the ortho-pyrophosphates, possesses 3D sodium diffusion pathways in the study crystal framework of a typical NASICON-type structure used as a cathode delivered a capacity of 113  and 108  mA·h·g^−1^ at 0.05C and 0.1C, respectively (1C = 120 mA·g^−1^). At 0.5C, the capacity was 80 mA·h·g^−1^ after 400 cycles, and at 20C, the capacity was still 60 mA·h·g^−1^ after 4400 cycles, which corresponds to a retention of 69.1%. (Figure 5) [115,116].

The main problem of the NASICON-based cathodes is the low capacity limited to ≈ 100 mA·h·g^−1^. This capacity can be increased by choosing other phosphates. In particular, Na_2_Fe_3_(PO_4_)_3_/carbon nanotube nanocomposite delivered a capacity of 143 mA·h·g^−1^, but the problem is shifted to the cycle ability, since the capacity was stable over 50 cycles. Partial substitution of Fe for Mn only results in a decrease of capacity [117]. Fluffy Na_0.67_FePO_4_/CNT nanocactus used as a cathode delivered the same capacity, also stable over 50 cycles [118].

Fluorophosphates as cathode materials exhibit similar capacities ≈ 100 mA·h·g^−1^ as NASICON, but they have a higher operational voltage so that the loss of energy density is smaller. For instance, orthorhombic Na_2_CoPO_4_F/C delivers a capacity of 107 mA·h·g^−1^ with a voltage plateau at 4.3 V [119]. Vanadium-based fluorophosphates also benefit from a high voltage (average potential of 3.7 V), owing to the enhanced inductive effect of the (PO_4_)^3−^ polyanion and the larger ionicity of the F-V bond [120]. The recent progress on sodium vanadium fluorophosphates has been reviewed in [121]. In particular, Qiu et al. [122] synthesized a core/double shell structured Na_3_V_2_(PO_4_)_2_F_3_@C nanocomposite through in situ coating of the carbon and the prepared particles were uniformly distributed in the mesoporous carbon framework. As a cathode for SIB, this composite delivered 125, 123, 121, 116, 100, 92, 84 and 63 mA·h·g^−1^ at 0.5, 1, 5, 10, 20, 30, 50 and 100C, respectively. After 5000 cycles at 50C rate, the capacity was 62 mA·h·g^−1^, which corresponds to a retention of 65%. Following this result, many works synthesized Na_3_V_2_(PO_4_)_2_F_3_@C compounds using other forms of carbon, including graphene, carbon nanofibers, and carbon nanotubes [123,124,125,126,127,128]. All of them demonstrated very good rate capability. Among them, in situ carbon nanofibers coating on Na_3_V_2_(PO_4_)_2_F_3_@C (NVPF@C) particles were obtained through chemical vapor deposition (CVD) by using Fe as the catalyst. The optimum ratio of NVPF@C to Fe was 5:100 [127]. The corresponding cathode tested at 20C over 5000 cycles delivered a capacity of 93.3 mA·h·g^−1^ with a capacity retention of 86.3% at more than 99.5% coulombic efficiency. Nanosized Na_3_(VOPO_4_)_2_F electrode also showed promising electrochemical properties, with a delivered capacity of 112 mA·h·g^−1^ with capacity retention of 93.8% after 200 cycles at a current rate of C/5, for an average discharge potential of 3.75 V leading to an energy density of 384 Wh·kg^−1^ At 2C, the capacity was still ≈ 100 mA·h·g^−1^ with retention of 90% over 1200 cycles [129]. A major progress was obtained recently by Wang et al. who fabricated an advanced low-T sodium-ion full battery assembled with this high-voltage cathode, and an anode of 3D Se/graphene composite [130]. This cell exhibited ultra-long lifespan (over 15,000 cycles, the capacity retention is still up to 86.3% at 1 A·g^−1^), outstanding low-*T* energy storage performance (e.g., all values of capacity retention are > 75% after 1000 cycles at temperatures from 25 to −25 °C at 0.4 A·g^−1^). At high current density of 4 A·g^−1^, the capacity is still 72.7 mA·h·g^−1^ at room temperature. Therefore, this cell well satisfies the requirements of grid energy storage for batteries. A flexible and binder-free Na_3_(VOPO_4_)_2_F cathode with nanocubes tightly assembled on carbon cloth was recently fabricated by a facile solvothermal method [131]. About 90% (112 mA·h·g^−1^) and 86% (106 mA·h·g^−1^) of the 1C capacity were retained at 10C and 20C, respectively, a rate performance superior to prior works, taking into account the high mass loading > 2.0 mg·cm^−2^. In total, 88% of the discharge capacity was retained after 1000 cycles while cycled at 5C.

### 2.5. Sulfates

Sulfates have raised interest, since alluaudite Na_2_Fe_3_(SO_4_)_3_ as a cathode material [132]. This cathode delivered a capacity of 100 mA·h·g^−1^, but with poor capacity retention; but most of all, the Fe^2+^/Fe^3+^ redox potential is raised at 3.8 V vs. Na^+^/Na, one of the highest among all the Fe-based intercalation compounds, owing to the electron-drawing (SO_4_)^2−^. This feature was the motivation for further investigation of sulfates of the same family: Na_2+2x_Fe_2-x_(SO_4_)_3_ [133,134,135], Na_2+2x_Mn_2-x_(SO_4_)_3_ or Na_2.5_(Fe_1-y_Mn_y_)_1.75_(SO_4_)_3_ [136,137,138] without improving the electrochemical properties. The hydrated sulfate compounds Na_2_Fe(SO_4_)_2_∙4H_2_O, Na_2_Fe(SO_4_)_2_∙2H_2_O [139], or eldfellite-type NaFe(SO_4_)_2_ [140] also gave poor results.

NaFe_3_(SO_4_)_2_(OH)_6_ is amorphous material that delivered a capacity of 120 mA·h·g^−1^ at low C-rate (C/20) with an average voltage of 2.72 V vs. Na^+^/Na in Na-ion cells [141]. However, the cycle life has been studied over 20 cycles only, and the rate capability has not been explored, yet. In an attempt to increase the capacity, the orthosilicate Na_2_FeSiO_4_ has been synthesized by electrochemical Li–Na ion-exchange. As it can theoretically exchange two Na^+^ ions, its use as an anode delivered a capacity of 330 mA·h·g^−1^ at a current density of 10 mA·g^−1^, but neither the cycle life nor the rate capability is good [142].

Alluaudite-type Na_2+2x_Fe_2-x_(SO_4_)_3_ has a low electronic conductivity which limited its electrochemical performance. Recently, however, graphene-Na_2+2x_Fe_2-x_(SO_4_)_3_ (NFS@rGO) microsphere composite was constructed via a facile spray-drying method in which the NFS particles were embedded in the three-dimensional (3D) graphene skeleton uniformly [143]. This composite delivered a capacity of 99 mA·h·g^−1^ at 0.1C and 78 mA·h·g^−1^ at 60C. It also demonstrated 80.8% capacity retention after 2000 cycles at 30C.

### 2.6. Pyrophosphates

Na_2_FeP_2_O_7_ crystallizes in the triclinic *P1* space group with 3D Na channels, and delivers a reversible capacity of 82 mA·h·g^−1^ with a redox potential of 3 V vs. Na^+^/Na [144,145]. With an ionic liquid electrolyte, a capacity retention of 91% after 1000 cycles was achieved [146]. High power and good cycle ability was also demonstrated with Na_4-x_Fe_2-x/2_(P_2_O_7_)_2_ (2/3 ≤ *x* ≤ 7/8), which is isostructural with Na_2_FeP_2_O_7_ [147], in particular, with Na_3.12_Fe_2.44_(P_2_O_7_)_2_. With this composition, however, a reaction of the electrolyte with sodium carbonate of the electrolyte results in the formation of a solid electrolyte interface (SEI), but this surface oxidation can be avoided by synthesis from an off-stoichiometric mixture of starting materials with a nominal composition of Na_3.42_Fe_2.44_(P_2_O_7_)_2.05_. The final composition in this case is the off-stoichiometric Na_3.32_Fe_2.34_(P_2_O_7_)_2_ that delivered a capacity of 85 mA·h·g^−1^ with coulombic efficiency of 99.0%, stable over 60 cycles that have been tested.

**Figure 6:**
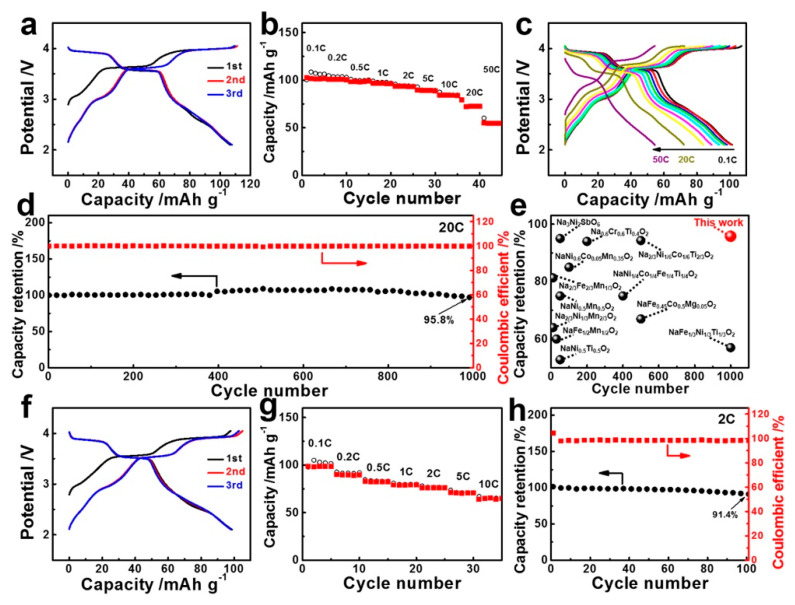
Electrochemical performance of P1-Na_2.3_Cu_1.1_Mn_2_O_7-δ_ (P1-NCM) in half cells and full cells. (**a**) Typical potential profiles of P1-NCM//Na at 0.1C rate. (**b**) Capability and (**c**) potential profiles of P1-NCM//Na at various current rates. (**d**) Cycling performance and Coulombic efficiency of P1-NCM//Na at 20C rate. (**e**) Summary of cycling stability for conventional layered metal oxide materials. (**f**) Typical potential profiles of P1-NCM//hard carbon at 0.1C rate. (**g**) Capability of P1-NCM//hard carbon at various current rates. (**h**) The cycling performance and Coulombic efficiency of P1-NCM//hard carbon at 2C rate. Reproduced with permission from [151]. Copyright 2018 Elsevier.

More impressive was the rate capability, since at a 10C rate, 72% of the reversible capacity at C/10 was still delivered. The authors attributed the fast kinetics to the spacious channel size along the *a*-axis, along with a single-phase transformation upon de/sodiation. This performance of pyrophosphates was achieved without carbon coating, nor any binder. Subsequent works have optimized the results by adding different carbon coatings, or modifying the deviation from stoichiometry [148,149]. These materials are of low cost and have a good thermal stability, and a very good cycle ability and rate capability [150], but their very small energy density has hindered their development. In particular, among P1 structure based cathode materials, their electrochemical performance is outperformed by manganese-based oxide with copper doping, i.e., Na_2.3_Cu_1.1_Mn_2_O_7-δ_ that surpassed most of the copper-doped cathode materials, with its energy density of 383 Wh·kg^−1^ (capacity of 106.6 mA·h·g^−1^, capacity retention 95.8% after 1000 cycles at 20C) and its average voltage of 3.6 V (Figure 6) [151].

V-based pyrophosphate compounds look more promising than Fe-based ones, even though their specific capacity is almost the same (80 mA·h·g^−1^), because their operating voltage is higher, which improves the energy density. The high redox potential is due to the inductive effect of the interaction between the transition metal and polyanions in the particular structure of these compounds where VO_6_ octahedra share every corner with a P_2_O_7_ group. As a result, this operating potential is 3.8 V for the V^4+^/V^5+^ redox reaction vs. Na^+^/Na in the case of Na_2_(VO)P_2_O_7_ [152], and even 4.13 V for the V^3+^/V^4+^ redox reaction in Na_4_V_3_(P_2_O_7_)_4_ [153]. In this last case, the material demonstrated 75% capacity retention after 600 cycles, owing to a very small change of volume (1%) during cycling.

### 2.7. Mixed Polyanionic Compounds

Synergetic effects resulting from the mixing of phosphates and polyphosphates have been explored. Na_4_Mn_3_(PO_4_)_2_(P_2_O_7_) as a cathode demonstrated a reversible capacity of 109 mA·h·g^−1^ at a rate of C/20 at the Mn^2+^/Mn^3+^ redox potential of 3.84 V vs. Na^+^/Na, demonstrating an energy density of 416 Wh·kg^−1^. [153]. Contrary to other Mn-based compounds with small cycle ability due to the JT distortion due to Mn^3+^, the cycle stability and the rate capability were good. The first-principle calculations showed that these features result from the low-activation-energy barriers of the three-dimensional Na diffusion pathways. Moreover, the JT distortion opens up sodium diffusion channels, contrary to the situation met in most manganese-based electrodes [154,155].

Na_4_Fe_3_(PO_4_)_2_(P_2_O_7_) (NFPP) integrates the advantages of both iron-based phosphates (NaFePO_4_) and pyrophosphates (Na_2_FeP_2_O_7_) but both ionic and electronic conductivities are very low. Recently, however, a facile spray-drying route was used to synthesize a NFPP@rGO composite in which NFPP particles with an average size of about 60 nm are homogeneously enwrapped by three-dimension (3D) interconnected rGO networks [156]. This material demonstrated a capacity of 128 mA·h·g^−1^ at 0.1C, an outstanding rate capability (35.1 mA·h·g^−1^ at 200C) and long cycling life (62.3% of capacity retention over 6000 cycles at 10C rate).

Na_7_V_4_(P_2_O_7_)_4_(PO_4_) operates at 3.88 V vs. Na^+^/Na, with a good cycle ability (capacity retention of 78% over 1000 cycles) [157]. When nano-structured, it can deliver 80% of the theoretical capacity at 10C rate and 95% of the initial capacity after 200 cycles [158]. The problem, however, is the small capacity (73 mA·h·g^−1^) which limits its practical application. In Na_3_*M*(CO_3_)(PO_4_) with *M* = Mn [159], or *M* = Fe [160], the capacity is higher (120–125 mA·h·g^−1^); but now, it is the poor rate capability that hinders their application.

### 2.8. Prussian Blue Analogs (PBAs)

**Figure 7:**
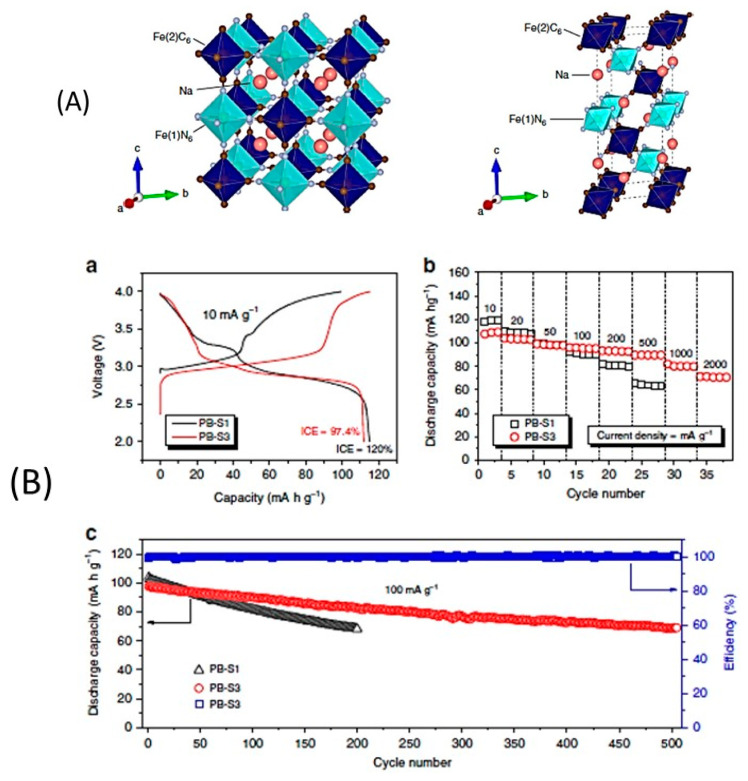
(**A**) Schematic structure of Na_2-x_FeFe(CN)_6_ (Na in red, C in brown, N in grey, Fe1 and Fe2 in cyan and blue, respectively). (**B**) The electrochemical properties. (**a**) initial charge–discharge curves, (**b**) rate and (**c**) cycling performance. Reproduced with permission from [167]. Copyright 2020 Springer.

These materials have been extensively investigated as cathodes for Na-ion cells, because their large alkali-ion channels enable fast kinetics with limited change of volume during cycling [161,162,163,164,165,166]. One difficulty with transition metal oxides is the strong bonding between oxygen anions (O2−) and the metal cation, which makes difficult the motion of the big Na^+^ ion. Hexacyano ion (C≡N)_6_^6−^ based electrode materials for Na-ion storage seems a better choice due to the weakened bonding between cyanide (C≡N)^−^ and cations. Moreover, the high-temperature calcination needed for many cathodes is not required during synthesis of PBAs, which lowers the manufacturing costs [166]. In addition, a simple and scalable co-precipitation method makes possible the synthesis of a Prussian-blue material that shows stable cycling performance over 1000 cycles (Figure 7) [167]. The general formula of PBAs can be described as *A*_x_*P*[*R*(CN)_6_]_1-y_□_y_∙*m*H_2_O (*A*: alkali metal ion; *P*: N-coordinated transition metal ion; *R*: C-coordinated transition metal ion; □: [*R*(CN)_6_] vacancy; 0 ≤ *x* ≤ 2; 0 ≤ *y* ≤ 1). In particular, when *P* = *R* = Fe, two types of Fe sites are present: one with a C end in the low-spin state (Fe1) and another with an N end in the high-spin state (Fe2). Therefore, when this material is used as a cathode material in sodium-ion cell, two voltage plateaus should be observed, corresponding to Fe1 and Fe2. In practice, however, only that of Fe2 is observed at 2.7 V, which reduces the capacity. To remedy this problem, Yang et al. synthesized a composite hexacyanoferrate-graphene oxide flakes, using a spray-drying method [168]. The coordinated water in the Prussian blue analog was removed during the co-heating of graphene oxide and the hexacyanoferrate. This removal of the coordinated water improved importantly the electrochemical properties since the Fe1 plateau could now be observed at 3.2–3.4 V and could contribute to the capacity. The best results obtained with Na_0.81_Fe[Fe(CN)_6_]_0.79_□_0.61_ (□ is a Fe(CN_6_ vacancy) plus 6.2 wt.% reduced graphene oxide. Used as a cathode, it delivered a capacity of 163.3 mA·h·g^−1^ at 30 mA·g^−1^ when cycled between 2.0 V and 4.0 V, and 112 mA·h·g^−1^ at a current density of 800 mA·g^−1^. A capacity retention of 91.9% (137.6 mA·h·g^−1^) was observed after 500 cycles at 200 mA·g^−1^. The removal of coordinated water is thus mandatory to obtain good electrochemical properties with Prussian blue analogs.

A dehydrated Na_2-δ_MnFe(CN)_6_·*m*H_2_O (δ ≈ 0.1; *m* ≈ 0.3) phase demonstrated a capacity of 150 mA·h·g^−1^ with an average voltage of 3.5 V and exhibited 75% capacity retention after 500 cycles [161]. Improved kinetics were obtained with highly crystalline NaFe_2_(CN)_6_/graphene composite. The capacity at low rate (150 mA·h·g^−1^ at 25 mA·g^−1^) was smaller than in [167], but the rate capability was improved with a capacity of 0.122 and 0.107 Wh·g^−1^ at rate of 1 and 2 A g^−1^, respectively [169]. This fast kinetics was attributed to the reduced concentration of Fe(CN)_6_ vacancies that hamper the electron transport along the CN framework. The slow crystallization process helped to obtain defect-free cubic particles tightly interconnected by the corrugated graphene oxide layers. Another strategy experimented to increase the capacity was the increase the concentration of sodium per formula. You et al. prepared Na-rich sodium iron hexacyanoferrate of composition Na_1.63_Fe_1.89_(CN)_6_ by controlling the reducing agent and reaction atmosphere during synthesis. Used as a cathode, it demonstrated a specific capacity of 150 mA·h·g^−1^ with 90% capacity retention after 200 cycles [170].

Another strategy was the synthesis of nanospheres with a hierarchical hollow architecture that can provide large numbers of active sites for sodium ions. These nanospheres demonstrated a capacity of 142 mA·h·g^−1^ [171]. The combination of Na-rich and multicomponent was also tried. Na_1.72_MnFe(CN)_6_ was obtained through introducing a large amount of NaCl [162], but the rate capability was small, due to the JT distortion due to Mn^3+^. Na_2_Ni_x_Co_1-x_Fe(CN)_6_ had a better rate capability but the capacity was below 100 mA·h·g^−1^.

Ref. [172]. Actually, better results were obtained without the substitution of Co for Ni; Na_2_CoFe(CN)_6_ nanocrystals demonstrated a capacity of 150 mA·h·g^−1^ and a ∼90% capacity retention over 200 cycles [173]. Na_1.76_Ni_0.12_Mn_0.88_[Fe(CN)_6_]_0.98_ delivered a capacity of 95 mA·h·g^−1^ with capacity retention of 83.8% after 800 cycles [174]. Recently, sodium full batteries derived from *X*-Fe (*X* = Co, Ni, Mn) Prussian blue analogs (PBAs)were investigated. In particular, Ni_0.67_Fe_0.33_Se_2_ coming from Ni-Fe PBAs showing core–shell structure in a dual-carbon matrix used as an anode retained an ultralong-term stability of 375 mA·h·g^−1^ after 10,000 cycles even at 10 A·g^−1^. The full cell using this anode vs. Ni-Fe PBA delivered a capacity of 302 mA·h·g^−1^ at 1.0 A·g^−1^ [175]. Table 1 lists the electrochemical properties of selected cathode-materials reviewed in the text.

## 3. Anodes

Anode materials are a key element to close the gap between lithium- and sodium-ion batteries, and have been subject to many investigations. A review with a primary emphasis on alloy anodes has been published in [176]. Recent progresses include other types of composites, starting with the conventional carbonaceous anodes, but also other composites based on the intercalation process and the alloying process.

### 3.1. Carbon

#### 3.1.1. Graphite

Utilization of pristine graphite as an anode for SIBs is difficult because of exfoliation during insertion of Na^+^ ions [177]. It is possible, however, to store sodium reversibly in graphite through co-intercalation reactions [178,179]. By this process, graphite anodes demonstrated cycle ability of thousands of cycles and high rate capabilities [180,181,182]. Until recently, the sodium co-intercalation voltage was in the range of 0.6−0.8 V vs. Na^+^/Na using various ether-based electrolytes [183,184]. Xu et al. decreased the reaction voltage down to 0.43 V for graphite-based half-cells, by adjusting the relative stability of ternary graphite intercalation compounds and the solvent activity in the electrolyte [185]. In particular, the full cell with Na_1.5_VPO_4.8_F_0.7_ cathode and 2 mol·L^−1^ NaPF_6_ dimethyl ether electrolyte demonstrated a power density of 3863 W·g^−1^ with an energy density of 112 Wh·kg^−1^ (based on the total mass of electrode materials). A capacity retention of 93% was also obtained after 1000 cycles at 1 A·g^−1^. Actually, graphitic carbon materials are now considered as promising for advanced sodium-ion batteries [186].

Al_2_O_3_ nanoclusters around 1 nm grown on the defects of a 3D porous graphene monolith suppress the decomposition of conductive sodium salt in the electrolyte, and reduce the detrimental etching of the SEI by hydrogen fluoride (HF). After introduction of Al_2_O_3_, higher initial Coulombic efficiency (ICE) of 70.2% and capacity retention of 82.9% after 500 cycles at 0.5 A·g^−1^ than those of normally reported for large surface area carbons were achieved. This new way to deactivate defects and improve the SEI is thus promising for the commercial use of carbon as anode materials for SIBs [187].

#### 3.1.2. Non-Graphitic Carbon

Hard carbon material can deliver 200 mA·h·g^−1^ at 25 mA·g^−1^ after 100 cycles [188], and a review of hard carbon-based negative electrodes for sodium ion batteries published before 2015 can be found in [189,190]. To obtain a good rate capability, nano-structured carbon is needed [191]. For instance, hollow carbon nanospheres prepared through the hydrothermal carbonization of glucose in the presence of latex templates delivered a capacity of 160 mA·h·g^−1^ was obtained at 100 mA·g^−1^ after 100 cycles [192]. Better results were obtained with hollow carbon nanowires prepared by pyrolyzation of hollow polyaniline nanowires, which delivered a capacity of 200 mA·h·g^−1^ at 125 mA·g^−1^ after 200 cycles [193]. Highly disordered carbon (HDC) synthesized by self-assembling of poly(diallyldimethylammonium chloride) (PDDA) and poly(sodium 4-styrenesulfonate) (PSS), and subsequent pyrolysis delivered a capacity of 225 mA·h·g^−1^ with a capacity retention of 92 % at 100 mA·g^−1^ after 180 cycles [194]. Electrospun carbon nano-fibers (CNFs) delivered a capacity of 233 and 82 mA·h·g^−1^ at 0.05 A·g^−1^ and 2 A·g^−1^, respectively, with 97.7% capacity retention after 200 cycles [195]. This result was attributed to their weakly ordered turbostratic structure and a large interlayer spacing between graphene sheets, which illustrates features of CNFs obtained by electrospinning. A review of carbon-based anodes obtained by this synthesis process has been published by Wang et al. [196]. Porosity is another parameter allowing for an increase of the electrochemical properties. A porous hard carbon synthesized by the pyrolysis of H_3_PO_4_-treated biomass (pomelo peels) delivered a capacity of 181 mA·h·g^−1^ at 200 mA·g^−1^ after 220 cycles and retained a capacity of 71 mA·h·g^−1^ at 5 A·g^−1^ [197]. However, the coulombic efficiency and cycle ability were damaged by the formation of a thick SEI due to side reactions with P. Apple biowaste was successfully used to synthesize a hard-carbon material for use as an anode in Na-ion cells that delivered capacity of 85 mA·h·g^−1^ in the 1000th cycle at 5C (1 A·g^−1^) [198]. The best results can be obtained by combining synergetic effects of porosity, N-doping and nano-structuration, under the form of nanosheets [199] or nanofibers [200,201], the corresponding anodes delivering a typical capacity of 220 mA·h·g^−1^ at 50 mA·g^−1^. Nitrogen-rich carbon with interconnected mesoporous structure could deliver a reversible capacity of 338 mA·h·g^−1^ at a current density of 30 mA·g^−1^, and remarkable rate capability with a capacity of 111 mA·h·g^−1^ at a current density of 500 mA·g^−1^ over 800 cycles [202]. Yang et al. reported that, at 0.15C (1C = 375 mA·g^−1^), N-doped carbon sheets delivered a stable reversible capacity of 292 mA·h·g^−1^. Even at high mass loading of 7.12 mg·cm^−2^ the reversible capacity was maintained at 121.7 mA·h·g^−1^. At 4.5C, the capacity was stable at circa 50 mA·h·g^−1^ over 2000 cycles [203]. N-doped carbon sheets were also investigated by Yang et al. to produce an anode delivering a capacity of 165 mA·h·g^−1^ after 600 cycles at current density of 200 mA·g^−1^ [204]. N/S co-doped ordered mesoporous carbon delivered a capacity of 419 mA·h·g^−1^ at 0.1 A·g^−1^ after 150 cycles, retaining 220 mA·h·g^−1^ at 5 A·g^−1^ even after 3000 cycles [205]. Although N-doping is most popular, doping with other elements such as B, O, S, and P are also of interest, and a review on the design, synthesis, and electrochemical properties of heteroatom-doped carbon anodes can be found in [206]. Recently, 3D scaffolding S-doped carbon nanosheets produced from biomass delivered a reversible capacity of 605 mA·h·g^−1^ at 50 mA·g^−1^, 133 mA·h·g^−1^ at 10 A·g^−1^. The capacity was maintained ats ~211 mA·h·g^−1^ upon 2000 cycles at current density of 5 A·g^−1^ [207].

Yun et al. synthesized pyroprotein-based carbon nanoplates (CNPs) with varying degrees of carbon ordering [208]. They showed that the sodium-ion storage mechanism varies from chemi-physisorption insertion to nanoclustering of metallic states, depending on the carbon structure of CNPs, which display various potentials and capacities. Therefore, tailoring carbon orderings is a critical factor for tuning the electrochemical performance of carbonaceous materials for SIBs. A perspective for sodiation of hard carbon consists of Na-ion storage at defect sites, by intercalation and last via pore-filling [209,210]. In addition, ab initio calculations for disordered carbon show that large initial interlayer distances and defects, in particular vacancies can greatly enhance the Na^+^ ion intercalation [211]. In addition, hard carbon can be used with ionic liquid electrolytes to obtain less-flammable sodium-ion cells [212]. Moreover, Li et al. demonstrated that the rate capability of hard carbon is underestimated in prior studies that used carbon/Na two-electrode half-cells, because it is the overpotential of the sodium counter electrode that drives the half-cells to the lower cutoff potential prematurely during hard carbon sodiation [213].

**Figure 8:**
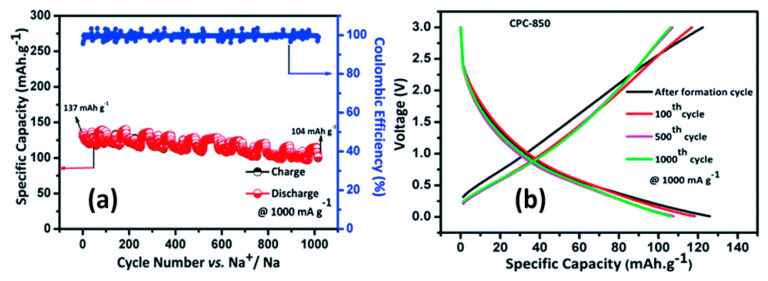
(**a**) Charge-discharge behavior of coir pith derived carbon prepared at 850 °C (CPC-850) as an anode at 1000 mA·g^−1^. (**b**) Voltage vs. capacity behavior CPC-850 anode upon progressive cycles after formation cycle. Reproduced with permission from [217]. Copyright 2017 Royal Society of Chemistry.

There many ways to synthesize carbon anodes. However, since the practical application of the sodium-ion technology relies on the fact that it is cost effective with respect to the Li-ion technology as recalled in the introduction, attention has been focused on of cheap, scalable and facile synthesis of the carbon anodes. In this respect, attention has been focused on carbonaceous materials derived from biomass waste. Most of the carbon materials derived from biomass exhibit specific capacity in the range of 200–300 mA·h·g^−1^ at a current density of 50 mA·g^−1^ in sodium-ion batteries [214,215,216]. In particular, a coir pith waste derived carbon (CPC) electrode demonstrated a capacity of 220 mA·h·g^−1^ up to 300 cycles with negligible capacity fading have been observed at 50 mA·g^−1^. Furthermore, CPC prepared at 850 °C delivers ∼110 mA·h·g^−1^ for 1000 cycles at 1 A·g^−1^ (Figure 8) [217]. Carbon materials derived from biomass also demonstrate a good performance as sodium-ion supercapacitor [218].

A simple productive synthesis of carbon quantum dots with diameters in the range of 1.5–3.0 nm was discovered by Hou et al. by mixing acetone and NaOH, without any other treatment [219]. An outstanding cycle life was demonstrated with a capacity of 150.1 mA·h·g^−1^ after 3000 cycles at current density 2.5 A·g^−1^. At 5 A·g^−1^, a capacity of 99.8 mA·h·g^−1^ was maintained after 10,000 cycles. Soft carbons have been less investigated, but can be competitive to hard carbon, provided that the precursor and heat treatment are optimized so that the interlayer distance *d* is large [220]. The best results were obtained for a soft carbon with *d* = 3.65 Å, which delivered a capacity of 120 mA·h·g^−1^ after 250 cycles at a current density of 1000 mA·g^−1^.

### 3.2. Metal Chalcogenide-Based Anodes

Metal oxides have also been extensively studied as anodes for sodium-ion batteries because of their low operating voltage vs. Na^+^/Na, and some of them have a high capacity [221]. They are divided in two families, depending on the intercalation or conversion reaction at the origin of their electrochemical properties.

#### 3.2.1. Intercalation-Based Materials

A huge effort of research has been devoted to the intercalation compounds [222]. Among them, TiO_2_ is abundant on earth and not toxic. Among the different polymorphs, the anatase phase looks more promising [223]. TiO_2_ has a high capacity, provided that the discharge cut-off voltage is decreased to 0.01 V vs. Na^+^/Na, in which case the capacity ≈ 193 mA·h·g^−1^ is achieved [224]. There has been a debate on the origin of the electrochemical activity of anatase TiO_2_. Kim et al. attributed the de/intercalation in to the redox activity of Ti^4+^/Ti^3+^ during charge/discharge, while Gonzalez et al. suggested pseudo-capacitive reactions [225]. Wu et al. clarified the origin of the storage mechanism owing to in situ XRD and ex situ XPS experiments on TiO_2_ nanoparticles [226]. They determined that the Ti^3+^:Ti^4+^ ratio is approximately 2.23, corresponding to 0.69 Na per TiO_2_ after discharge, while it decreases to 0.35 after charge, corresponding to 0.28 Na per TiO_2_ remaining in the structure (intercalation reaction). In addition, the reduction of TiO_2_ to metallic Ti along with the structural rearrangement (conversion reaction) is observed. In conclusion, the chronological electrochemical process is the following: (i) pseudo-capacitive reaction during the initial discharge process; (ii) structural rearrangement; (iii) disproportionation reaction and formation of Ti^0^ and O_2_ during further discharge; (iv) reversible Na de-insertion occurring in Na_x_(TiO_2_) (0.28 ≤ *x* ≤ 0.69). In any case, TiO_2_ is not a good electrical conductor, so that is must be nano-structured. The 3D array architecture is particularly suited to obtain large accessible surface and yet maintains short ion-transport distance [227]. However, the electrochemical activity of TiO_2_ arrays might be compromised by the low surface reactivity, in which case surface functionalization is a key approach in the realization of high electrochemical activity [228,229,230]. Ni et al. combined the 3D nanotube architecture with phosphate functionalization [231]. The surface phosphorylated TiO_2_ nanotube arrays (noted P-TiO_2_) were obtained by electrochemical anodization of Ti metal in NH_4_F solution and subsequent phosphorylation using sodium hypophosphite. Another advantage is that the self-supported configuration eliminates the need for a binder and conducting additive so that the P-TiO_2_ nano-arrays can be directly adapted as an electrode. As a result, this electrode afforded a reversible capacity of 334 mA·h·g^−1^ at 67 mA·g^−1^ (0.2C) and a superior rate capability. At 3350 mA·g^−1^ (10C) the electrode delivered a capacity of retains a capacity of 143 mA·h·g^−1^ over 500 cycles and 141 mA·h·g^−1^ (≈ 94% of that in the 2nd cycle) over 1000 cycles. This result illustrates that the construction of binder-free and self-supporting electrodes improves the reaction kinetics and electrode stability. The reason is that it avoids the polymer binder/conductive additives that may cause virtual swelling in common electrolytes and result in a poor electrochemical performance [232]. Avoiding the binder may also be beneficial to relax the volume expansion. Another example is provided by TiO_2_ nanorods grown on carbon fiber cloth as binder-free electrode grown by a facile hydrothermal method [233]. As an anode for SIBs, it exhibited an exceptional electrochemical performance, including excellent rate capability and cyclic stability, maintaining a high capacity of 148.7 mA·h·g^−1^ after 2000 cycles at 1 A·g^−1^.

Composites with conductive carbon helps to improve the electronic conductivity and thus the electrochemical properties, in particular the rate capability. In particular, graphene is the most conductive form of carbon and has thus been considered for this purpose [234,235,236]. A graphene-TiO_2_ composite delivered a capacity of 115 mA·h·g^−1^ at a current of 1 A·g^−1^ and a stable specific capacity of 102 mA·h·g^−1^ at 0.1 A·g^−1^ after 300 cycles. [237]. Chen et al. synthesized a graphene-coupled TiO_2_ sandwich-like hybrid (10 wt.% graphene) in which intercalation pseudocapacitance dominated the charge storage process [238]. At a current density of 500 mA·g^−1^ (∼2C), after the initial dozens of cycles, this composite delivered a reversible capacity of 120  mA·h·g^−1^ kept unchanged during the subsequent 4300 cycles. The rate capability was also excellent, with a reversible capacity of 90 mA·h·g^−1^ at an extremely high current density of 12,000  mA·h·g^−1^.

Other forms of carbon have been used successfully to coat the TiO_2_ particles. Combining the synergetic effects of a small size (11 nm) of TiO_2_ particles and uniform carbon coating to improve the conductivity of the powder, carbon coated anatase TiO_2_ particles delivered a capacity of 134 mA·h·g^−1^ at 10C (3.35 A·g^−1^) and 1227 mA·h·g^−1^ at 0.1C, with high cycling stability (full capacity retention between 2nd and 300th cycle at 1C) and high coulombic efficiency (≈ 99.8%) [239]. Carbon coated anatase TiO_2_ hollow spheres prepared through the carbon wrapping of etched amorphous TiO_2_ solid spheres demonstrated a capacity of capacity of 140.4 mA·h·g^−1^ after 500 cycles at 5C rate, and 84.9 mA·h·g^−1^ after 80 cycles at 25C [240].

We know from Hou et al. that carbon quantum dots can be used as anodes for sodium-ion batteries [219]. In a subsequent work, this group designed a hierarchical anatase TiO_2_ homogeneously tuned by using carbon through Ti–C bonds, exploiting carbon quantum dots as uniform carbon additives with surface area (202 m^2^·g^−1^) and abundant mesopores. The corresponding anode delivered a high reversible specific capacity of 264 mA·h·g^−1^ at a rate of 0.1C (33.6 mA·g^−1^) and still maintains 108.2 mA·h·g^−1^ even after 2000 cycles at 10C with a retention of 94.7% [241]. Carbon dots were also used to decorate N-doped TiO_2_ nanorods [242]. Utilized as an anode, this composite delivered a capacity of 185 mA·h·g^−1^ with 91.6% retention even at a high rate of 10C over 1000 cycles. Inverse opal TiO with N-doped carbon layer and oxygen vacancies surface as an anode material for sodium-ion battery delivered a capacity of 140 mA·h·g^−1^ after 400 cycles under 1 A·g^−1^, owing to a pseudo-capacitive contribution of 73.38% at 1 mV·s^−1^ [243].

**Figure 9:**
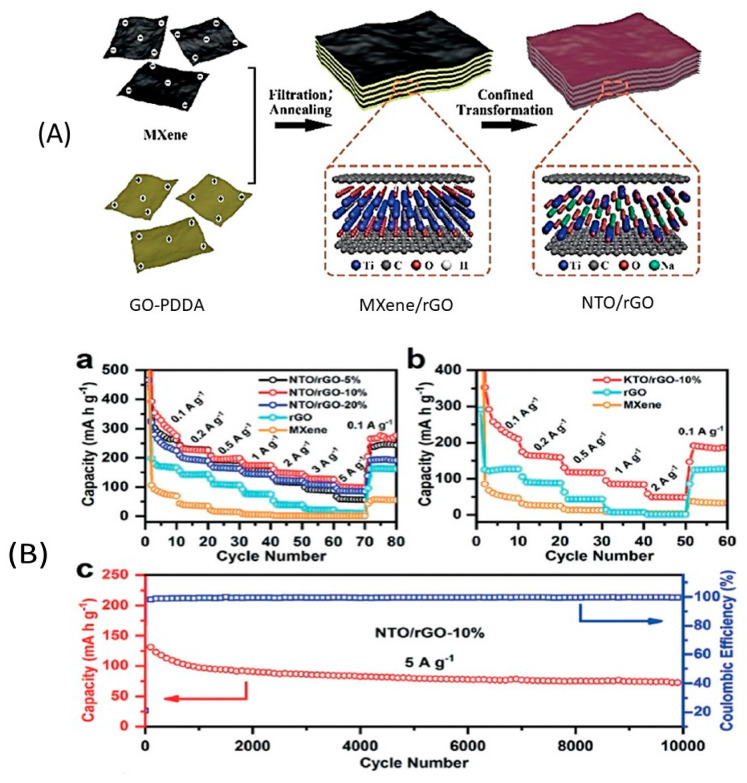
(**A**) Synthesis of free-standing films of sodium titanate nanosheets (NTO) sandwiched between graphene layers from *MX*ene (titanium carbide) and reduced graphene oxide (rGO) nanosheets. (**B**) Electrochemical performance of the NTO/rGO films for SIBs. (**a**) Comparison of rate performance of NTO with different rGO content, rGO and *MX*ene at various current densities. (**b**) Comparison of rate performance of KTO/rGO-10 %, rGO and *MX*ene at various current densities. (**c**) Long-term cycling discharge capacities and Coulombic efficiencies of NTO/rGO-10 % at 5 A·g^−1^. Reproduced with permission from [245]. Copyright 2016 Elsevier.

The performance of TiO_2_ depends, like any electrochemically active material, on its porosity and structure. This can be evidenced by the performance of TiO_2_ mesocages with high surface area (204 m^2^·g^−1^) and uniform mesoporous structure. A capacity of 93 mA·h·g^−1^ (per gram of TiO_2_) was retained after 500 cycles at 10C in the range of 0.01–2.5 V [244]. In that case, the active particles were not a composite, only TiO_2_ particles, but they were admixed with polyvinylidene fluoride (PVDF) binder and acetylene black carbon additive in a weight ratio of 70:20:10 to form the anode. The performance of titanate also depends very much on the type of carbon additive. The incorporation of graphene into the titanate films produced efficient binder-free anodes delivering a reversible capacity of 72 mA·h·g^−1^ at 5 A·g^−1^ after 10,000 cycles (Figure 9) [245].

#### 3.2.2. Conversion Reaction Compounds

Conversion reaction compounds are attractive because their capacity of Na-storage is larger than that of intercalation compounds. However, it is more difficult to overcome the deterioration of the material upon cycling due to the change of volume and structure. Nevertheless, progress has been done in the recent years, and transition metal oxides are now considered as potential active elements for sodium ion batteries [246,247,248,249,250,251,252,253,254,255,256,257,258,259,260,261,262,263,264,265,266,267,268,269,270]. As a result, while carbon-based anode/TM-oxides cathode architectures were identified as the most promising in terms of energy density, the alloying-conversion anode/NASICON cathode geometry were identified as the most performing in terms of power density [36].

**a. Iron oxides**

**Figure 10:**
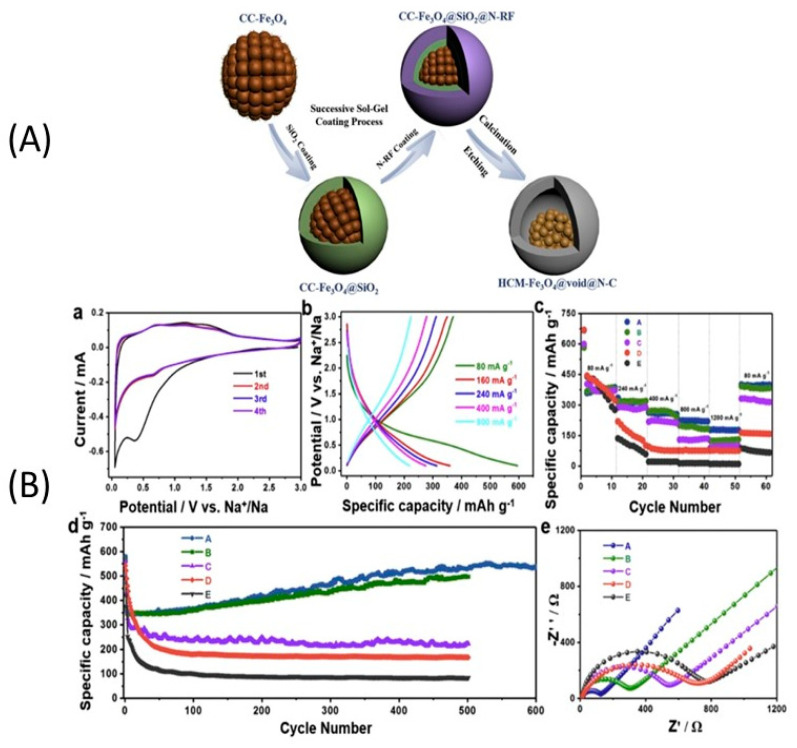
(**A**) Illustration for the synthesis of the yolk-shell structured highly crystallized mesoporous Fe_3_O_4_ in hollow N-doped Carbon Nanospheres (HCM-Fe_3_O_4_@void@N-C). Step 1: The citrate-capped Fe_3_O_4_ (CC-Fe_3_O_4_) nanoparticles were obtained through the solvothermal method. Step 2: The CC-Fe_3_O_4_ nanoparticles were successively coated by SiO_2_ and nitrogen-doped resorcinol-formaldehyde (N-RF) through sol-gel method. Step 3: The HCM-Fe_3_O_4_@void@N-C were produced *via* calcining the CC-Fe_3_O_4_@SiO_2_@N-RF nanospheres and etching the SiO_2_ layer through hot NaOH solution. (**B**) The cyclic voltammetry curves (**a**) of the initial four cycles obtained within a voltage range of 0.01–3.0 V and charge-discharge curves (**b**) at different current densities of the yolk-shell structured HCM-Fe_3_O_4_@void@N-C nanospheres. The rate capabilities at different current densities (**c**), the cycling performance at 160 mA·g^−1^ (**d**), and the Nyquist plots (**e**) of A HCM-Fe_3_O_4_@void@N-C, B HCM-Fe_3_O_4_@void@C, C HCM-Fe_3_O_4_@C, D HCM-Fe_3_O_4_, and E CC-Fe_3_O_4_ nanospheres. Reproduced with permission from [259]. Copyright 2015 Elsevier.

Fe_3_O_4_ is of great interest due to its high theoretical capacity, low cost, and abundance on earth. Core-shell nano-structured Fe_3_O_4_@carbonaceous composites can avoid the pulverization of the particles owing to the robust carbon that also improves the kinetics as it is a good electrical conductor. In particular, Liu et al. strongly bound homogeneously dispersed Fe_3_O_4_ quantum dots with an average size of 3.8 nm onto hybrid carbon nanosheets. The corresponding anode for sodium-ion cell delivered a capacity of 286–416 mA·h·g^−1^ at 0.1–2.0 A·g^−1^. At current density of 1.0 A·g^−1^, a capacity of 252 mA·h·g^−1^ was still obtained after 1000 cycles [258]. Mesoporous Fe_3_O_4_@nitrogen-doped carbon yolk-shell structured nanospheres were synthesized via sol-gel coating routes and confinement calcination strategy (Figure 10) [259]. As an anode, this composite delivered an outstanding capacity of capacity of 522 mA·h·g^−1^ after 800 cycles at 160 mA·g^−1^.The results obtained with Fe_2_O_3_-reduced graphene oxide (RGO) are less impressive, but have been obtained with a more scalable synthesis process (a facile microwave-assisted reduction of graphene oxide in Fe_2_O_3_ precursor) [260]. The composite with 30 wt.% RGO demonstrated a capacity of 289 mA·h·g^−1^ at a current density of 50 mA·g^−1^ after 50 cycles [261]. Inter-connected nanochannels and *γ*-Fe_2_O_3_ nanoparticles (5 nm) uniformly embedded in a porous carbon matrix were synthesized via an aerosol spray pyrolysis technique. As an anode, this composite delivered the discharge capacity of 740 mA·h·g^−1^ after 200 cycles. The capacity remained at 317 mA·h·g^−1^ at a current density of 8000 mA·g^−1^. Fe-ZIF derived Fe_2_O_3_ embedded in the nitrogen-doped carbon matrix with strong oxygen-bridge bonds demonstrated a capacity of 473.7 mA·h·g^−1^ at the current density of 100 mA·g^−1^ after 100 cycles. The capacity remained at 155.3 mA·h·g^−1^ at 4 A·g^−1^ [262].

**b. Cobalt Oxides**

Cobalt-based compounds are among the most investigated materials for sodium storage [263]. For Co_3_O_4_, the complete reaction is based on the conversion mechanism [264]:

Co_3_O_4_ + 8Na → 3Co + 4Na_2_O. (1)

It possesses a high theory capacity of 890 mA·h·g^−1^, which justifies a lot of investigations. In the charge process, Co nanoparticles are partially oxidized to Co_3_O_4_, the other part of the CoO remain leading capacity loss [265]. Meso-porous cobalt oxide-based anode (Co_3_O_4_ mass loading of 0.6–0.8 mg·cm^−2^) for SIB was tested in 1 mol·L^−1^ NaPF_6_ in 1:1 (vol.%) fluoroethylene carbonate (FEC): Anhydrous diethyl carbonate (DEC) optimized electrolyte. It retained 80% of its maximum 204 mA·h·g^−1^ capacity at current density of 445 mA·g^−1^ through 200 cycles (and retained 75% capacity through 250 cycles) with near 100% coulombic efficiency [266]. As any material that operates via conversion reaction, nano-structuring and porosity are mandatory to alleviate the change of volume during cycling. Yang et al. used mesoporous silica as the template for the generation of dual porosity Co_3_O_4_ with spherical mesopores and porous nanochannels. The dual porosity mesopores allow better transport pathways and increase the effective surface area in contact with the electrolyte. Consequently, this Co_3_O_4_ electrode delivered an initial capacity of 707 mA·h·g^−1^ at a current density of 90 mA·g^−1^, retaining a capacity of 416 mA·h·g^−1^ after 100 cycles [267]. The synergetic effect of Co_3_O_4_ nanoparticles and conductive carbon has been explored with different kinds of carbon including carbon nanotubes [268,269], graphene [270] and N-doped graphite (NC) [271]. This Co_3_O_4_/NC hybrid with flower-like structure as an anode for SIB delivered a capacity of 214 mA·h·g^−1^ after 100 cycles at 0.1 A·g^−1^, excellent rate capability (145 mA·h·g^−1^ at 2 A·g^−1^ and 130 mA·h·g^−1^ at 4 A·g^−1^) and long-term cycling stability (120 mA·h·g^−1^ after 2000 cycles at 0.5 A·g^−1^). Porous hollow Co_3_O_4_ with N-doped carbon coating (Co_3_O_4_/N-C) polyhedrons delivered a capacity of 229 mA·h·g^−1^ within 150 cycles at 1 A·g^−1^ [272]. Various shapes have also been investigated: nanosheets [273,274,275], nanocubes [276], shale-like [277], yolk–shell dodecahedrons [278], flower-like [271] with similar results. We can then conclude that the experimental capacity reported in the literature is far below the theoretical one. One of the largest reversible capacity has been obtained 523.5 mA·h·g^−1^ after 50 cycles at rate of 25 mA·g^−1^ in the voltage range of 0.01–3 V vs. Na^+^/Na on mesoporous Co_3_O_4_ sheets/3D graphene networks nanohybrids [279]. Unfortunately, the investigation of the cycle ability has been tested on 50 cycles only. Rambutan-like hybrid hollow spheres of carbon confined Co_3_O_4_ nanoparticles synthesized by a facile one-pot hydrothermal treatment delivered a capacity of 712 mA·h·g^−1^ at a current density of 0.1 A·g^−1^, and 223 mA·h·g^−1^ at 5 A·g^−1^. It also demonstrated 74.5% capacity retention after 500 cycles [280]. In an attempt to increase the cycle life Co_3_O_4_/metal oxide heterostructures were synthesized. An example is the graphene/SnO_2_/Co_3_O_4_ (GSC) heterojunction [281]. Consequently, this graphene oxide/SnO_2_/Co_3_O_4_ anode achieved a reversible capacity of 461 mA·h·g^−1^ after 80 cycles at a current density of 0.1 A·g^−1^. At a high current density of 1 A·g^−1^, a high reversible capacity of 241 mA·h·g^−1^ after 500 cycles was demonstrated. Co_3_O_4_ is a p-type semiconductor while SnO_2_ is an n-type semiconductor. In the discharge process, the internal electric field then points from the SnO_2_ side to the Co_3_O_4_ side as an effective p-n junction. As a result, a depletion region is formed, reducing the accumulation of charge at the interfaces, which is favorable to the diffusion and insertion of Na^+^ ions. Other heterostructures have been synthesized. For example, carbon-encapsulated wire-in-tube Co_3_O_4_/MnO_2_ heterostructure nanofibers (Co_3_O_4_/MnO_2_@C) synthesized via electrospinning followed by calcination delivered 306 mA·h·g^−1^ at 100 mA·g^−1^ over 200 cycles, but also showed a cycling stability of 126 mA·h·g^−1^ after 1000 cycles at a high current density of 800 mA·g^−1^ [282]. We can also cite ZnO/Co_3_O_4_ [283].

**c. Copper Oxide**

As an anode for Na storage, CuO is a promising material as a result of its high abundance, and high theoretical capacity of 674 mA·h·g^−1^ [284]. This high capacity corresponds to the possibility to store 2 Na^+^ ions and form Cu and 2Na_2_O. Actually, binder-free porous CuO rod arrays grown on Cu foil delivered a capacity of 640 mA·h·g^−1^ at a high current density of 200 mA·g^−1^ [285]. Real-time microstructural evolution during the sodiation of CuO nanowires revealed that the sodiation process consists of three steps. First, Cu_2_O and Na_2_O were predominantly formed; then, the intermediate NaCuO phase is nucleated; the final sodiation products are Na_6_Cu_2_O_6_, Na_2_O and Cu [286]. CuO nanoparticles (∼10 nm) homogeneously embedded in the carbon matrix with a carbon weight of 44% as an anode for SIB delivered a capacity of 402 mA·h·g^−1^ after 600 cycles at a current density of 200 mA·g^−1^, and the capacity maintained at 304 mA·h·g^−1^ at 2 A·g^−1^ [287]. This composite was synthesized through aerosol spray pyrolysis.

**d. Tin Oxide**

Tin oxide can be used as an anode material for SIBs, owing to the reactions:

SnO_2_ + 4Na^+^ + 4e^−^ → Sn + 2N_2_O, (2)

*x*Na^+^ + Sn + *x*e^−^ ↔ Na_x_Sn. (3)

The first reaction is irreversible (first reduction process), the second one is reversible. 3D porous carbon encapsulated SnO_2_ nanoparticles composite delivered a high reversible specific capacity of 280 mA·h·g^−1^ after 250 cycles at a current density of 100 mA·g^−1^. This composite demonstrated a capacity of 100 mA·h·g^−1^ after 1000 cycles even at a high current density of 1600 mA·g^−1^ [288]. A SnO_2_/C nanocomposite consisting in dispersed SnO_2_ nanoparticles on super P carbon spheres used as an anode delivered a discharge capacity of 293 mA·h·g^−1^ after 100 cycles at a current density of 50 mA·g^−1^, and 150 mA·h·g^−1^ at 1000 mA·h·g^−1^ [289]. The oxygen vacancies-containing amorphous SnO_2_ ordered arrays retained the capacities of 376 mA·h·g^−1^ after 100 cycles at 0.05 A·g^−1^ and 220 mA·h·g^−1^ after 800 cycles at 1 A·g^−1^. The capacity was maintained at 200 mA·h·g^−1^ at 20 A·g^−1^ [290]. One-dimensional (1-D) ordered nanoporous SnO_2_ nanostructures vertically assembled on a Cu substrate were prepared by an efficient method that combines cold-rolling and anodization [291]. This electrode combines the synergetic effects of being binder free to optimize the electrochemical properties and porosity to buffer the change of volume during cycling. It delivered a high capacity of 326 mA·h·g^−1^ over 200 cycles at a current rate of 0.2 C.

**e. Sulfides**

Both cobalt sulfides and selenides are investigated as active anode materials for Na-ion batteries. However, selenium is rare and almost 40 times more expensive than sulfur. Therefore, the substitution of S for Se will cancel the main advantage of the SIBs with respect to the LIBs, namely their lower cost, as mentioned in the introduction. We then report in this review only the results obtained on metal sulfides. For selenides, we guide the reader to recent reviews [263,292].

N-rich carbon-coated Co_3_S_4_ ultrafine nanocrystal (Co_3_S_4_@NC) exhibiting ultrafine nanocrystals with a diameter of about 5 nm delivered a capacity of 420.9 mA·h·g^−1^ at the current density of 100 mA·g^−1^ after 100 cycles, indicating that the cycling performance is strengthened by the nitrogen-doped carbon coating. The capacity was maintained at 284 mA·h·g^−1^ at 1 A·g^−1^. These good results were attributed to the porous structure inherited from the zeolitic imidazolate framework-67 (ZIF-67) precursor [293].

**Figure 11:**
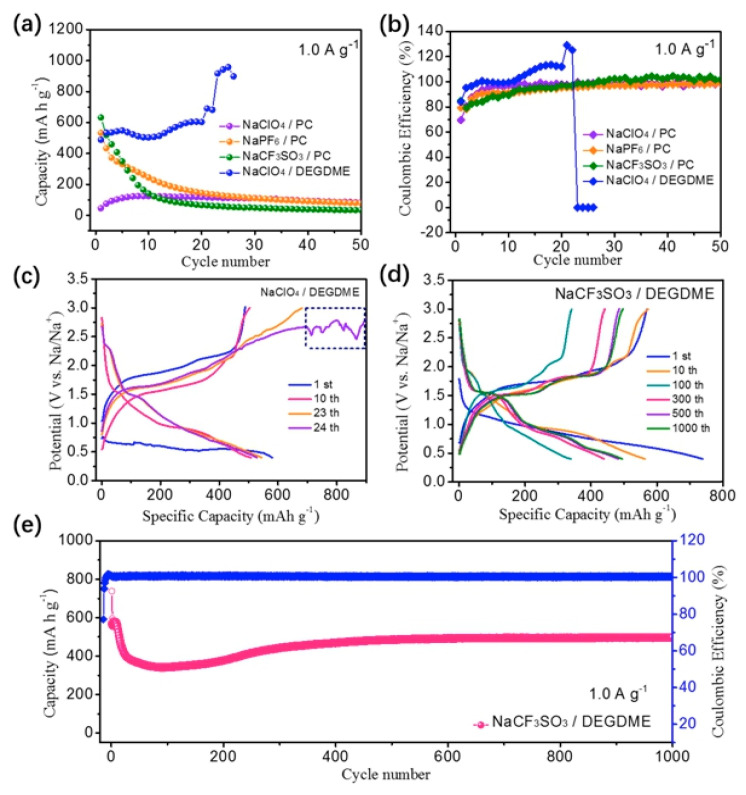
(**a**,**c**) Cycling performances and (**d**) coulombic efficiency of the anode composed of NiCo_2_S_4_ nanodots (9 nm) and N-doped carbon in different electrolytes at the current density of 1.0 A·g^−1^. The galvanostatic discharge-charge profiles in (**b**) NaCF_3_SO_3_/DEGDME and (**e**) NaClO_4_/DEGDME electrolytes. Reproduced with permission from [296]. Copyright 2011 Wiley.

In parallel with Co_3_S_4_, the thiospinel NiCo_2_S_4_ has been subject recently to a lot of attention. It has a high electrical conductivity at room temperature (1.25 × 10⁶ S·m⁻^1^), two orders of magnitude higher than that of the oxide counterpart (NiCo_2_O_4_). This property can be used to obtain extremely rapid kinetics by introducing the pseudocapacitance effect into Na-ion batteries, so that this bimetallic sulfide deserves a special attention. Zhao et al. synthesized hexagonal nanosheets with a large lateral dimension of ~2 μm and thickness ~30 nm through coprecipitation followed by a vapor sulfidation method [294]. As the anode material in SIBs, these NiCo_2_S_4_ nanosheets delivered a reversible capacity of 387 mA·h·g⁻^1^ after 60 cycles at a current density of 1000 mA·h·g^−1^. The sodium ion storage process was a result of a combined Na^+^ intercalation and conversion reaction between Na⁺ and NiCo_2_S_4_, plus the contribution of the pseudocapacitance mechanism increasing with the current density, as large as 71% at a scan rate of 0.4 mV·s⁻^1^. A composite composed of an RGO matrix and a hollow prism of NiCo_2_S_4_ with a typical size of 500–600 nm as an anode for SIB demonstrated a capacity of 530 mA·h·g^−1^ with negligible fading after 70 cycles at 50 mA·g^−1^. At current density of 800 mA·g^−1^, the capacity was 220 mA·h·g^−1^ [295]. NiCo_2_S_4_ nanodots (~9 nm) uniformly incorporated with N-doped carbon delivered a capacity of 570 mA·h·g^−1^ after 200 cycles at 0.2 A·g^−1^, and still retains 395 mA·h·g^−1^ at 6 A·g^−1^ after 5000 loops. The choice of the electrolyte, important to obtain such a result, was the ether-based electrolyte NaCF_3_SO_3_/DEGDME to promote faster sodium-ion transportation due to flexible one-dimensional chain structure and favorable solvent-salt interaction in the voltage region 0.4–3.0 V (Figure 11) [296].

Co_9_S_8_ suffers a conversion reaction according to:

Co_9_S_8_ + 16Na^+^ → 9 Co + 8 Na_2_S, (4)

which shows a relatively high theoretical capacity of 544 mA·h·g^−1^ [297]. Higher rate capability and higher cycle ability with this material is obtain with an ether-based electrolyte, such as 1 mol·L^−1^ sodium trifluomethanesulfonate (NaCF_3_SO_3_) salt dissolved in tetraethylene glycol dimethyl ether (TEGDME) [298].

Co_9_S_8_ quantum dots (3 nm in size) embedded into porous carbon frameworks were obtained using SiO_2_ as sacrificial template [299]. Owing to the combination of macroporosity (average size 150 nm), the carbon that helps to maintain the structural integrity and improves the rate capability by increasing the electrical conductivity, and the nano-structuration, the corresponding anode demonstrated a capacity of 340 mA·h·g^−1^ after 2000 cycles with the Coulombic efficiency of over 99%, at a current density of 1 A·g^−1^. At high current density of 10 A·g^−1^, the capacity was still maintained at 253 mA·h·g^−1^. Utilizing a Co-based metal-organic framework (MOF) as the precursor, in particular the zeolitic imidazolate framework [300] is another effective way to synthesize a hierarchical Co_9_S_8_-based material. In particular, a Co_9_S_8_ quantum dot/hollow carbon matrix/graphene aerogel synthesized with the ZIF-67 precursor delivered a capacity of 628 mA·h·g^−1^ after 500 cycles at a current density of 300 mA·g^−1^ and demonstrated an exceptional rate capability with a capacity of 330 mA·h·g^−1^ at 6400 mA·g^−1^ [301]. A yolk–shell structured Co_9_S_8_/MoS_2_ polyhedron with N-doped carbon composite, synthesized through a step by step process using again ZIF-67 as the precursor, delivered a capacity of 438 mA·h·g^−1^ within 150 cycles at a current density of 1.0 A·g^−1^, and 421 mA·h·g^−1^ within 250 cycles at a high current density of 2.0 A·g^−1^ [302]. These remarkable electrochemical properties were attributed to the MoS_2_ shell that brings a lower activation energy for Na^+^ ion diffusion and larger exposed surface to the electrolyte, and also improves the electrical conductivity.

MoS_2_ is one of the most promising anode materials for SIBs, provided that the huge variation of volume during cycling is alleviated. In addition, its electronic conductivity is small. To overcome these problems, the strategy is always the same: fabricate nano-structured composites with conductive carbon. MoS_2_-graphene composites, MoS_2_-CNT hybrids, and MoS_2_-carbon spheres have been tested with significant improvement in the electrochemical properties [303,304] The best performance was achieved recently with a MoS_2_@CNFIG composite, where CNFIG stands for a carbon nanofiber interpenetrated graphene architecture. This anode delivered a capacity of 598  mA·h·g^−1^ at 0.1 A·g^−1^ based on the total mass of MoS_2_ and CNFIG matrix. At 1 A·g^−1^, the capacity was still 412 mA·h·g^−1^ in the 1000th cycle, which corresponds to a capacity retention of 86.2% based on its initial specific capacity (478 mA·h·g^−1^) in the 2nd cycle. The rate capability was also remarkable, with a capacity of 366 mA·h·g^−1^ achieved at 5 A·g^−1^ after 1000 cycles, achieving capacity retention of 86.9% [305].

CoS_2_ can react with four Na^+^ ions and this electrochemical reaction can be divided into 2 steps. Firstly, when the voltage is above 1 V *vs*. Na^+^/Na, no more than two Na^+^ ions inserted into CoS_2_ and an intermediate product is formed. Then, when the voltage is decreased below 1 V, Co^4+^ is reduced to metallic Co and Na_2_S emerges. To buffer the volume change of CoS_2_ during cycling, composites were synthesized with different forms of carbon: multi-wall carbon nanotubes (MWCN) [306], reduced graphene oxide [307], graphene and carbon nanotubes [308]. The best results were obtained with the CoS_2_-MWCN anodes with a capacity maintained at 568 mA·h·g^−1^ after 100 cycles (69% of first discharge capacity) at current density of 0.1 A·g^−1^ in NaCF_3_SO_3_-DGM electrolyte. Note the results are very sensitive to this choice of electrolyte, since poor results were observed for the same composite in NaClO_4_-EC/PC. Using 1 mol·L^−1^ NaCF_3_SO_3_ in diethylene glycol dimethyl ether (DEGDME) as the electrolyte, CoS_2_ nanoparticles wrapping on flexible freestanding multichannel carbon nanofibers delivered a similar capacity at low current rate (537 mA·h·g^−1^ at 0.1 A·g^−1^), but in addition, a remarkable rate capability and cycle ability was demonstrated with a capacity of 315 mA·h·g^−1^ at 1 A·g^−1^ after 1000 cycles. Even at 10 A·g^−1^, the capacity was maintained at ~202 mA·h·g^−1^ [309]. Pan et al. synthesized flower-like N-doped carbon/CoS_2_ spheres (N-C/CoS_2_) by a solvothermal method followed by sulfurization [310]. Owing to conductive interconnected wrinkled nanosheets that create mesoporous structures, and many extra defect vacancies and Na^+^ storage sites introduced by the nitrogen doping process, the results were outstanding. This anode delivered a capacity of 698 at 1 A·g^−1^ after 500 cycles, and the capacity was still 458 mA·h·g^−1^ at 10 A·g^−1^. The same group fabricated an anode with Double-Morphology (nanoparticle and nanosheet) CoS_2_ Anchored on N-doped multichannel carbon nanofibers (CoS_2_@MCNFs). CoS_2_ nanosheets were in situ formed on the surface of the carbon fiber, while CoS_2_ nanoparticles also grew in the channels of these carbon fibers. With this unique structure, the carbon nanofiber plays the dual roles of matrix and coating for CoS_2_ nanosheets and nanoparticles, respectively. This anode delivered a capacity of 620 mA·h·g^−1^ after 900 cycles at current density of 1 A·g^−1^, and 508 mA·h·g^−1^ can still be kept at 5 A·g^−1^ [311]. Similar to Co_9_S_8_, heterostructures consisting of CoS_2_ and other metal sulfides have also been reported: SnS_2_@CoS_2_-rGO [312], NiS_2_@CoS_2_@C@C [313]. In this last case, 600 mA·h·g^−1^ capacity was demonstrated after 250 cycles, at a current density of 1 A·g^−1^.

CoS has also been considered as an anode for SIBs. The electrochemical reaction can be divided into the insertion step:

CoS + *x*Na^+^ + *x*e^−^ → Na_x_CoS, *x* < 2, (5)

and the conversion step:

Na_x_CoS + (2 − *x*)Na^+^ + (2 − *x*)e^−^ → Co + Na_2_S. (6)

The conversion step takes place below 0.8 V [314,315,316]. As usual, the problem is to avoid the pulverization of the particles due to the big change of volume in the conversion reaction. One can always increase the lower voltage to avoid the conversion reaction to obtain an anode with good capacity retention, but in that case the capacity associated to the conversion reaction is lost and the energy density in a full cell with such an anode will be too small. For this purpose, Zhou et al. synthesized CoS nanoparticles embedded into porous carbon rods [317]. In case the CoS particles were 7 nm in diameter, this anode delivered a capacity of 542 mA·h·g^−1^ after 2000 cycles in the voltage range of 0.6–3 V vs. Na^+^/Na, with a capacity retention of 91.4% at 1 A·g^−1^) and demonstrated an excellent rate performance (discharge capacities of 510 mA·h·g^−1^ at 5 A·g^−1^ and 356 mA·h·g^−1^ even at 40 A·g^−1^). A full Na-ion cell with this anode and Na_3_V_2_(PO_4_)_3_ cathode exhibited a capacity of 352 mA·h·g^−1^ at 0.5 A·g^−1^. It should be noted that this small size of the particles was crucial, since the same anode synthesized with CoS particles with a diameter of 18.5 nm gave degraded electrochemical properties. Another strategy is the fabrication of a CoS@C yolk-shell microsphere composite, but the corresponding anode has been tested over 50 cycles only [318]. Carbon-coated Co–Sn–S hollow nanocubes synthesized through a solvothermal sulfuration show excellent rate performance (478 mA·h·g^−1^ at 10 A·g^−1^) owing to a pseudocapacitance-dominated sodium storage mechanism, but a moderate cycle ability (83% capacity retention after 100 cycles at 0.1 A·g^−1^) [319].

A recent regain of interest in antimony sulfide is due to the electrochemical performance of Multi-shell hollow structured Sb_2_S_3_ obtained from the ZIF-8 framework. In the first step, multi-shell ZnS particles were obtained after three quenching and sulfidation processes. Then, the multi-shell structured Sb_2_S_3_ microparticles were obtained via a simple ion-exchange method [320]. Used as an anode, they delivered a capacity of 909 and 604 mA·h·g^−1^ at the current densities of 100 and 2000 mA·g^−1^, respectively. After 50 cycles, the multi-shell Sb_2_S_3_ could still maintain a reversible capacity of over 500 mA·h·g^−1^ (against 200 mA·h·g^−1^ for the single shell Sb_2_S_3_). The high capacity is due to the high efficiency of the conversion reaction:

Sb_2_S_3_ + 6Na^+^ + 6e^−^ ⇄ 2Sb + 3Na_2_S, (7)

which enhances the alloying/dealloying reaction:

2Sb + 6Na^+^ + 6e^−^ ⇄ 2Na_3_Sb. (8)

In addition, the pseudocapacitive contribution raises from 44% to 84% under a sweep rate raising from 0.2 to 10 mV·s^−1^ due to the multi-shell structure that offers both the exterior and interior surfaces for the electrochemical reaction.

Iron sulfides FeF_2_ (pyrite), Fe_1-x_S (pyrrhotite), and FeS have also been considered as promising anode materials for SIBs [58]. FeS@C, FeS_2_@C, and FeS_2_/graphene anodes have been constructed, demonstrating that the carbon shell of the introduction of graphene was able to buffer the volume change during cycling and also improve the electrical conductivity and the C-rate [321,322,323,324,325]. Nanostructured FeS_2_ (50–80 nm) embedded in an N-doped carbon nanosheet composite (FeS_2_/CNS) *via* a combined template method and a solid state sulfuration method exhibited high specific capacity (812 mA·h·g^−1^ at 0.1 A·g^−1^), long cycling life (77.2% capacity retention after 350 cycles at 1 A·g^−1^) and excellent rate capability (400 mA·h·g^−1^ 5 A·g^−1^) [325]. Liu et al. fabricated flexible Fe_1-x_S-filled porous carbon nanowires/reduced graphene oxide (Fe_1-x_S@PCNWs/rGO) hybrid paper in which the PCNWs encapsulated with in situ formed Fe_1-x_S nanoparticles (NPs) were evenly dispersed between the rGO nanosheets [326]. As an anode, this composite delivered a capacity of 573–89 mA·h·g^−1^ over 100 consecutive cycles at 0.1 A·g^−1^ with areal mass loadings of 0.9–11.2 mg·cm^−2^ and high volumetric capacities of 424–180 mA·h·cm^−3^ in the current density range of 0.2–5 A·g^−1^. This is a remarkable result, since the mass loading is a very important parameter for commercialization, and is usually small for SIBs (< 0.5 mA·h·cm^−2^) [327], compared with that of lithium-ion batteries today (2–3 mA·h·cm^−2^).

Another iron sulfide of interest as an active element of anode SIBs is Fe_7_S_8_. Its theoretical capacity is 662 mA·h·g^−1^ according the reaction [328]:

Fe_7_S_8_ + 16Na^+^ + 16e^−^ → 7Fe + 8Na_2_S. (9)

Moreover, its charge-discharge peaks of cyclic voltammetry after the first cycle (reduction peak at 0.92 V corresponding to the reaction between Na^+^ and Na_2-x_FeS_2_; oxidation peak at 1.38 V) [329] is lower than that of FeS_2_ (cathodic peaks at 1.2, 1.6 and 2.1 V, anodic peaks at 1.5, 2.0 and 2.5 V [330], which is beneficial for the energy density of full cells. Another advantage comes from the fact that Fe_7_S_8_ is a semi-metal. Despite the higher conductivity, the association with graphite is beneficial to the electrochemical performance, because it makes possible to increase the loading. The flexible anode 3D carbon-networks/Fe_7_S_8_/graphene of Chen et al. [330] used a flexible anode with areal mass loading of 3 mg cm^−2^ demonstrated a high areal capacity (2.12 mA·h·cm^−2^ at 0.25 mA·cm^−2^) and excellent cycle stability of 5000 cycles (0.0095% capacity decay per cycle). Copper sulfides have less been investigated. Nevertheless, promising results were obtained with CuS-RGO composite obtained by microwave-assisted reduction [331].

SnS nanoparticles anchored on three-dimensional N-doped graphene [332]. Oriented SnS nanoflakes were bound on S-doped N-rich carbon nanosheets by a hydrothermal method demonstrated high-rate capability (250.7 mA·h·g^−1^ at 20 A·g^−1^) and stable capacity retention (∼98% after 100 cycles at 1 A·g^−1^) as a SIB anode, with a dominating supercapacitance contribution [333]. Free-standing SnS/C nanofibers prepared by electrospinning used as an anode for SIBs retained a capacity of 481 mA·h·g^−1^ after 100 cycles at 50 mA·g^−1^, and 349 mA·h·g^−1^ at 200 mA·g^−1^ after 500 cycles [334]. Hollow ZnS-SnS@C nanoboxes encapsulated by graphene delivered a stable capacity of 302 mA·h·g^−1^ after 500 cycles at 500 mA·g^−1^ [335].

SnS_2_ embedded in nitrogen and sulfur dual-doped carbon nanofibers were synthesized using a facile electrospinning technique by Xia et al. [336]. The capacity of the corresponding anode remains at 380 mA·h·g^−1^ at 500 mA·g^−1^ after 200 cycles. At high current density of 4 A·g^−1^, the capacity was still 310 mA·h·g^−1^. The intercalation of Ni into the van der Waals gap of SnS_2_ exhibited an initial high reversible capacity of 795 mA·h·g^−1^ at 0.1 A·g^−1^, with a stable capacity retention of 666 mA·h·g^−1^ after 100 cycles. At a current density of 1 A·g^−1^, the capacity was 437 mA·h·g^−1^ [337]. To improve the coulombic efficiency caused by the partial irreversible conversion reaction of SnS_2_, Ou et al. fabricated heterostructured SnS_2_/Mn_2_SnS_4_/carbon nanoboxes by a facial wet-chemical method. Utilized as an anode, this composite delivered an initial capacity of 841 mA·h·g^−1^ with high ICE of 90.8%, excellent rate capability (488 mA·h·g^−1^ at 10 A·g^−1^) and delivered a capacity of 522 mA·h·g^−1^ at 5 A·g^−1^ after 500 cycles [338]. The SnS_2_/Mn_2_SnS_4_ heterojunctions were thus efficient to stabilize the reaction products Sn and Na_2_S. Wang et al. [339] synthesized SnS_2_ nanosheet arrays on a carbon paper, with a preferential (001) edge orientation, which facilitates rapid electrochemical reaction kinetics with preferential edge orientation. At current density of 50 mA·g^−1^, this binder-free anode delivered discharge and charge capacities of 1056 and 647 mA·h·g^−1^, respectively. After this irreversible loss of capacity associated to the formation of the SEI, the coulombic efficiency was better than 98%, and a capacity of 631 mA·h·g^−1^ was retained after 150 cycles.

**Figure 12:**
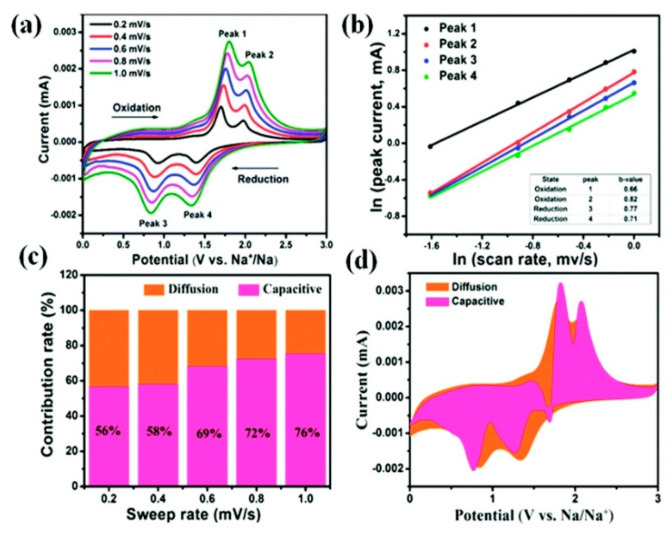
Electrochemical properties of NiS_2_ particles (diameter ≈ 5 nm) embedded in porous carbon nanofibers (NiS_2_NP/p-CNF), as an anode. (**a**) Cyclic voltammograms at different scan rates; (**b**) the fitted lines of ln(*i*) *versus* ln(*v*) plots in different oxidation and reduction states; (**c**) The contribution ratio of capacitance at different scan rates (0.2, 0.4, 0.6, 0.8, 1 mV#xB7;s^−1^) of NiS_2_NP/p-CNF; (**d**) the area comparison of capacitive contribution to total storage in CV at 1 mV#xB7;s^−1^. Reproduced with permission from [343]. Copyright 2017 Royal Society of Chemistry.

NiS [340] and NiS_2_ [341] have attracted interest. In particular, NiS_2_ is cheap and its theoretical capacity is 873 mA·h·g^−1^ as it proceeds via a four-electron conversion reaction over the sodiation/desodiation process [342]. Porous NiS_2_ nanoparticles 5 nm in thickness, embedded in porous carbon nanofibers was synthesized by an electrospinning process accompanied by further sulfide treatment [343]. As an anode, it delivered a capacity of 500 mA·h·g^−1^ at 0.1 A·g^−1^, 200 mA·h·g^−1^ at 2.0 A·g^−1^. When cycled at this high current density of 2.0 A·g^−1^, the capacity maintained at 120 mA·h·g^−1^ after 2000 cycles (Figure 12).

**f. Cobalt phosphide**

Recent progress has been also made on CoP-based anodes [344]. CoP nanoparticles (11.3 nm in diameter) uniformly embedded in N-doped carbon nanosheets (CNSs) were fabricated via the simple one-step calcination of a Co-based metal–organic framework (MOF) and red phosphorous. [345]. The composite delivered a Na-storage capacity of 598 mA·h·g^−1^ at 0.1 A·g^−1^ according to the total mass of the composite, i.e., 831 mA·h·g^−1^ per gram of CoP, and demonstrated a long-term stability with 98.5% capacity retention after 900 cycles at 1 A·g^−1^. The capacity at low rate is thus close to the theoretical one (890 mA·h·g^−1^) expected from the conversion reaction [346]:

CoP + 3Na^+^ + 3e^−^ ⇄ Co + Na_3_P. (10)

This result illustrates the interest of the use of MOFs as templates, and a comprehensive review of MOF-derived nanostructures as anodes for LIBs and SIBs can be found in [347]. It also demonstrates that the side reaction:

Na_3_P → P + 3 Na^+^ + 3e^−^, (11)

during the charge could be avoided, as this reaction alters the performance of the anode for two reasons: part of the phosphorous does not recombine with Co to form CoP, and P is a bad electrical conductor [348]. Another difficulty is the huge change of volume (500%) during sodiation-desodiation, implying that nano-structuration and synthesis of composites are mandatory. CoP nanowires were formed by growing cobalt carbonate hydroxide hydrate Co(CO_3_)_0.5_OH·0.11H_2_O on carbon paper substrate. Then, the CoP wires were coated with polypyrrole by a simple in-situ polymerization process [349]. The 5-nm thick polypyrrole coating layer was able to buffer the change of volume of the CoP nanowires (50 nm in diameter), and the carbon paper was used as a conductor. Consequently, the anode delivered an areal capacity of 0.443 mA·h·cm^−2^ at 1.5 mA·cm^−2^ after the first cycles, without capacity fading over 1000 cycles. At the high current density of 3 mA·cm^−2^, the discharge capacity of 0.285 mA·h·cm^−2^ was maintained. Like in the case of the other cobalt compounds already reported above, ZIF-67 is commonly used as the precursor to obtain dispersed nano-sized particles, while the organic framework converts to conductive carbon framework by carbonization. In particular, it was utilized to synthesize core/shell structured CoP@C polyhedrons anchored 3D reduced graphene oxide on nickel foam CoP@C-RGO-NF [350]. This binder-free anode demonstrated a capacity of 473 mA·h·g^−1^ at a current density of 100 mA·g^−1^ after 100 cycles.

**g. Intermetallic Compounds**

Group-15 elements (P, As, Sn, Bi) can serve as functional alloying elements for SIBs. They have in common a high theoretical capacity, because of the possibility to take multiple Na^+^ ions per single atom with an average voltage of less than 1 V. The counterpart is that the catch of multiple Na^+^ ions is accompanied by a large volume expansion and a strong structural stress since the radius of Na^+^ is larger than that of Li^+^. Like in the case of conversion reactions, the difficulty is thus to maintain the structural stability and the cycle life. That is why these materials are associate to another composite element like carbon to overcome this problem. However, these material benefit from the particular ultrathin SEI formed with the use of ether-based electrolytes, enabling higher electrochemical performance [351].

*(i) Tin*. The theoretical capacity of Sn alloying up to Na_15_Sn_4_ is 847 mA·h·g^−1^ [352,353,354,355,356,357]. The encapsulation of Sn particles by carbon nanospheres under CVD conditions forming unique deflated Sn@C nanoparticles firmly attached on the surface of the 3-D carbon derived from walnut shell membranes was made by Chen and Deng [354]. The first cycle discharge (Na insertion) capacity was 260 mA·h·g^−1^, and the first cycle charge (Na extraction) was 163 mA·h·g^−1^ at 10 mA·g^−1^, but decreases fast with the number of cycles. Better results were obtained with C/Sn/Ni/TMV1cys, binder-free composite electrode, where TMV1cys stands for a novel mutant of tobacco mosaic virus (TMV) created via genetic engineering, wherein a cysteine codon is expressed within the N-terminus of coat proteins. These tin-coated viral nanoforests as anodes retained a capacity of 405 mA·h (g_Sn_)^−1^ after 150 deep cycles at current density of 50 mA·g^−1^ [355]. Similar results were obtained with a Sn-Cu nanocomposite that delivered a capacity of 420 mA·h·g^−1^ at 0.2C rate, retaining 97% of their maximum observed capacity after 100 cycles [356]. The best results, however, were obtained with a Sn–C composite (58 wt.% Sn and 42 wt.% N-doped carbon) in which 5–50 nm spherical Sn particles are on nitrogen-doped graphite nanoplatelets [357]. This anode delivered 429 mA·h (g_Sn+C_)^−1^ at 0.2C and most of all maintained 290 mA·h (g_Sn+C_)^−1^ after 1000 cycles at 1 A·g^−1^ (i.e., 82.6% retention in capacity referring to the second cycle (350 mA·h (g_Sn+C_)^−1^). This exceptional cycle life for a Sn-based anode was attributed to the effective electrode expansion reduced to 14% during sodiation, compared with 420% expected for Sn. Note, however, that the large amount of carbon reduces the effective capacity of the electrode. We have given many examples showing the advantage of the construction of binder-free electrodes, whenever it is possible. With metals, however, this is difficult because the huge volume change associated with the conversion reaction makes difficult the adhesion of the nano-arrays to the substrate upon cycling. Ni et al. reported recently a strategy of strengthening the connection between the electrode (Sn) and the current collector (Cu) by thermally alloying Sn and Cu at their interface region [356]. The locally formed tin-copper alloys served as a structural glue to guarantee the adhesion between Sn nanowall-shaped arrays and the Cu substrate, and the gradient-like distribution of Sn–Cu ensured no abrupt change in volume expansion/contraction during cycling so that it maintained the overall structural integrity over long cycles. This as-built binder-free electrode demonstrated a reversible capacity of 801 mA·h·g^−1^ at 0.2 C, a rate capability of 610 mA·h·g^−1^ at 5 C, and a retention of 501 mA·h·g^−1^ at 5 C after 300 cycles. This result demonstrates that this strategy is efficient and opens the route to the construction of binder-free anodes for SIBs with metals as the active element.

 (ii) Antimony. According to the reaction between Sb and Na_3_Sb, the theoretical capacity is 660 mA·h·g^−1^ [358,359] To approach this result and relieve the stress caused by the three Na uptake to obtain a good cycle ability, the synthesis of nanocomposite with different forms of carbon has been investigated. 1D carbon nanofibers, which trap Sb nanoparticles via a simple electrospinning process delivered a capacity of 631 mA·h·g^−1^ at C/15, 337 mA·h·g^−1^ at 5C, and demonstrated a good rate capability (90% capacity retention after 400 cycles at C/3) [360]. Other forms of carbon include acetylene black [361] and porous carbon [362]. In this last case, a capacity of 385 mA·h·g^−1^ (capacity retention of 88.5%) after 500 cycles at 100 mA·h·g^−1^ was demonstrated. Antimony/nitrogen-doping porous carbon (Sb/NPC) composite with polyaniline nanosheets as a carbon source delivered a capacity of 529.6 mA·h·g^−1^, with 97.2% capacity retention after 100 cycles at 100 mA·g^−1^ [363]. Binding Sb nanoparticles in ionic liquid-derived nitrogen-enriched carbon (Sb@NC) via pyrolysis of an SbCl_3_/1-ethyl-3-methylimidazolium dicyanamide mixture improved the sodium storage [364]. This anode delivered a capacity of 440, 285 and 237 mA·h·g^−1^ at a current density of 0.1, 2 and 5 A·g^−1^, respectively. At the current density of 100 mA·g^−1^, the capacity maintained at 328 mA·h·g^−1^ after 300 cycles. An electrode material composed of Sb nanoplates on Ni nanorod arrays exhibited a capacity of 580 mA·h·g^−1^ at a current density of 0.5 A·g^−1^ with 80% retention over 200 cycles [365]. The full cell with P2-Na_2/3_Ni_1/3_Mn_2/3_O_2_ as the cathode delivered a capacity of 580 mA·h·g^−1^ over 200 cycles and an energy density as high as 100 Wh·kg^−1^. These results show that antimony is one of the best-performing anode materials in terms of both capacity and cycling stability. This is surprising since the theoretical sodium-storage capacity of silicon is 954 mA·h·g^−1^, In practice, Si has never reached such a capacity. However, by combining silicon and antimony amorphous films with bilayer thickness down to 2 nm, and an amount of Si of 7 at.%, the mesoporous Si_0.07_Sb_0.93_ reached a capacity of 663 mA·h·g^−1^ after 140 cycles at a low rate of 20 mA·g^−1^. This is more than the theoretical capacity for Sb (660 mA·h·g^−1^) and more than the highest experimental capacity for pure Si reported so far (∼600 mA·h·g^−1^) [366]. Additional results for metallic Sn- and Sb-anodes can be found in [367].

(iii) Phosphorous. The theoretical capacity according to the reaction between P and Na_3_P is 2596 mA·h·g^−1^, and phosphorous has aroused growing interest as an anode element for sodium-ion batteries [368]. The allotropes of interest for SIBs are the red and the black phosphorous. The black-phosphorous is a good conductor (∼300 S·m^−1^), and the interlayer channel size is large (3.08 Å), so that Na^+^ ions of radius 2.04 Å can be stored between the phosphorene layers. Few phosphorene layers sandwiched between graphene layers shows a specific capacity of 2440 mA·h·g^−1^ (calculated using the mass of phosphorus only) at a current density of 0.05 A·g^−1^ and 83% capacity retention after 100 cycles while operating between 0 and 1.5 V [369]. This very high capacity was attributed to a dual mechanism of intercalation of sodium ions along the *x* axis of the phosphorene layers followed by the formation of a Na_3_P alloy that accompany the P-P bond breaking, in agreement with theoretical calculations [370]. Poly(3,4-ethylenedioxythiophene) (PEDOT) functionalized on surface-modified black-phosphorous nanosheets delivered a capacity of 1078 mA·h·g^−1^ at a current density of 0.1 A·g^−1^ after 100 cycles. The capacity delivered at higher rate were 750 (1 A·g^−1^) and 370 mA·h·g^−1^ (10 A·g^−1^) [371]. A black phosphorus/Ketjenblack–multiwalled carbon nanotubes (BPC) composite with 70 wt.% phosphorus content was fabricated by high energy ball milling [372]. This composite delivered a capacity of 1700 mA·h·g^−1^ after 100 cycles at 1.3 A·g^−1^ based on the mass of P. More recently, 4-nitrobenzene-diazonium-modified P was used to bond chemically with RGO to enhance the electrical connection between two species [373]. The additional functional groups enlarged the channels of the modified black phosphorous with RGO layers, thus improving the rate capability. A capacity of 650 mA·h·g^−1^ at 1 A·g^−1^ over 200 cycles was obtained with this composite. The drawback, however, was a capacity at low rate (1400 mA·h·g^−1^ at 0.1 A·g^−1^) smaller than the best results that can exceed 2000 mA·h·g^−1^: 2060 mA·h·g^−1^ at 0.2 C, with capacity retention of 75.3% after 200 cycles for a composite of black phosphorus and multiwall carbon nanotubes (BP–CNT) prepared *via* a surface oxidation-assisted chemical bonding procedure [374], 2119 and 1700 mA·h·g^−1^ at 0.2 and 1.3 A·g^−1^, respectively, over 100 cycles for a black phosphorous/Ketjenblack-MWCNTs composite [372]. Red phosphorous has also a high sodium storage theoretical capacity (2595 mA·h·g^−1^), but it shows lower electronic conductivity (≈ 10^−14^ S·cm^−1^), so that it must be associated to a conductive material to fabricate performing anodes for SIBs [375]. In addition, the volume expansion upon sodiation is large, so that hollow and porous structure has been fabricated to increase the cycle life. For instance, wet-chemical synthesis of hollow red-phosphorus nanospheres with porous shells used as an anode delivered 1364 mA·h·g_em_^−1^ and 1100 mA·h·g_em_^−1^ (g_em_ = gram of electrode materials) at 0.2C. The corresponding areal capacity was 2.3 and 1.8 mA·h·cm^−2^ at 0.52 and 1.3 mA·cm^−2^, respectively. At 1C, a stable capacity of 969.8 mA·h·g^−1^ was demonstrated over 600 cycles [376] Combining electroless deposition with chemical dealloying to control the shell thickness and composition of a red phosphorus (RP)@Ni–P core@shell nanostructure, Liu et al. obtained an anode with remarkable properties: 1256 mA·h·g^−1^ after 200 cycles at 260 mA·g^−1^, while at the high current density of 5.2 A·g^−1^, the capacity was 491 mA·h·g^−1^ retained at 409 mA·h·g^−1^ after 2000 cycles (the data are per gram of the composite) [377]. Hybridization of red phosphorous with functional conductive polymer-sulfurized polyacrylonitrile (P–SPAN) was obtained via a facile mechanical ball-milling process. The hybridization enabled an intimate contact of SPAN and the red phosphorous, which greatly improved the conductivity and helped forming a robust electrode that can endure large volume change upon cycling [378]. The corresponding anode delivered a capacity of 1300 mA·h·g^−1^ at 520 mA·g^−1^, with 91% capacity retention after 100 cycles. By confining nanosized amorphous red P into ZIF-8-derived nitrogen-doped microporous carbon matrix (denoted as P@N-MPC), Li et al. obtained an anode with a capacity of ≈ 600 mA·h·g^−1^ at 0.15 A·g^−1^ and improved rate capacity (≈ 450 mA·h·g^−1^ at 1 A·g^−1^ after 1000 cycles with a capacity fading rate reduced to 0.02% per cycle) [379]. Red phosphorous encapsulated into the cube shaped sandwich-like interconnected porous carbon building via the vaporization–condensation method demonstrated a capacity retention of about 93% at 2 A·g^−1^ after 100 cycles, and a capacity of 502 mA·h·g^−1^ at 10 A·g^−1^ [380].

(iv) Silicon. The large interstitial sites of amorphous Si (a-Si) facilitates Na intercalation and migration with respect to crystalline Si. Amorphous Si (a-Si) can theoretically absorb 0.76 Na atom per Si, corresponding to a specific capacity of 725 mA·h·g^−1^ [381]. In addition, the full sodiation process only induces a volume expansion of 114%, much less than that of other alloying materials like Sn, P, Sb. Nevertheless, the first attempts showed poor rate capability. More recently, however, an anode based on the rolled-up amorphous Si nano-membranes capacity of 152 mA·h·g^−1^ after 2000 cycles, corresponding to a capacity retention of ≈ 85% owing to a large supercapacitance contribution [382]. Crystalline Si is electrochemically active toward sodium storage through an amorphization mechanism of NaSi alloys [383]. In this work, Zhang et al. fabricated a flexible binder-free bamboo-rattle type Si/carbon nanofiber film via an electrospinning technology. As an anode, this composite delivered 454 mA·h·g^−1^ after 200 cycles at a current rate of 50 mA·g^−1^. The contribution of the carbon nanofibers was 157 mA·h·g^−1^, the remaining part coming from the 20 wt.% silicon. At a higher current density of 5 A·g^−1^, the capacity maintained at ≈ 200 mA·h·g^−1^ after 2000 cycles. Table 2 lists the electrochemical properties of selected anode materials reviewed in the text.

### 3.3. 3D Structuring

The large volume expansion of the alloy electrode during cycling is the key factor affecting its performance. A well-known strategy that is commonly used today is the construction of electrodes in which the carbon matrix can function as a stress-buffer during the volume expansion/contraction process of active materials and thus greatly enhance the mechanical stability of the overall electrode. Graphene has been widely used as a 2D sp^2^ carbon for this purpose. However, 3D graphene architecture with stable backbone demonstrates superiorities in restraining the severe aggregation of graphene sheets [384,385,386]. This concept, however, was mainly restricted to sulfides and oxides anode materials, because S and O bind closely to the functional groups of graphene. It does not extend easily to metals, because the contact between metal particles and graphene only relies on the weak van der Waals force, so that the detachment of metal particles from graphene during cycling is inevitable. In addition, the pre-preparation of 3D graphene architecture usually requires high cost, long reaction times and/or use of a toxic reducing agent for graphene oxide reduction, so the process is not scalable. A major progress has been obtained by Quin et al. who combined the industrialized spray drying method with space-confined catalysis effect, and developed a facile top-down strategy for the preparation of SnSb-in-plane nano-confined 3D N-doped porous graphene network [387]. This synthesis is featuring as a single continuous process without any pre-preparation of 3D graphene architecture and templates, and is thus not only less expensive, but also scalable. The reason for choosing SnSb alloy (Sn: 50 at%) rather than Sn or Sb was motivated by the fact that Sn has the ability of catalyzing the pyrolysis of carbon, and form graphene coating on its surface, while the volume expansion upon going through Sb (181.1 Å^3^) to hexagonal Na_3_Sb (237 Å^3^) is small, and SnSb metal is expected to inherit the advantages of both Sn and Sb. The SnSb nanocrystals (10–15 nm) were all-around coated by few-layer graphene (3–5 layers, ≈ 1 nm). As a result, this anode for SIB delivered a capacity above 400 mA·h·g^−1^ from the 2nd to 100th cycle at 0.1 A·g^−1^. At a high current density of 10 A·g^−1^, a capacity of ≈ 190 mA·h·g^−1^ was delivered and kept for 4000 cycles, inducing a capacity retention of nearly 100%. This is the best high-rate cycling performance ever reported for Sn/Sb-based materials. For comparison, the performance of other selected SnSb Na-ion battery anodes can be found in [388]. Wang et al. proposed a self-assembly NaCl template-assisted in situ catalytic strategy for preparing monodisperse multicore–shell SnSb@SnO_x_/SbO_x_@C nanoparticles (10–30 nm) space-confined in three-dimensional (3D) graphene-like porous carbon networks. As an anode for SIB, it delivered a capacity of 244 mA·h·g^−1^ at 5 A·g^−1^ and demonstrated a capacity retention of 80% after 500 cycles at 2 A·g^−1^ [389].

## 4. Conclusions

Considering cathodes, the successful reports of Novasis Energies, Inc. and Faradion Ltd. demonstrate the potential of Prussian blue analogs and Na_x_*M*O_2_ as SIB cathodes in stationary and large-scale energy storage applications. Owing to their large alkali-ion channels enabling fast diffusion of Na^+^ ions, Prussian blue analogs have excellent electrochemical properties. Moreover, they are not expensive. However, in a recent discussion on the cathode choice between layered transition metal oxides versus Prussian blue analogs for commercialization of SIBs, Liu et al. concluded that P2-type Na_x_*M*O_2_ and O3-type materials demonstrate better practical advantages [390]. Among polyanionic compounds, the recent progress on NASICON-based materials make them very attractive as cathodes for SIBS, with very good rate capability and capacity retention. However, polyanionic compounds are difficult to grow with uniform morphology and composition. They also suffer from their rather small capacity, which stays in the vicinity of 100 mA·h·g^−1^. Larger capacities can be achieved with the O3-type layered materials, in particular, iron- and manganese-containing oxides. In fact, these oxides benefit not only larger capacities, but also larger redox potential vs. Na^+^/Na, which also contributes to increase the energy density. They demonstrate realistic commercial perspectives for SIBs, based on good air stability, electrochemical performance, and low manufacturing expenditure. Their rate capability is smaller than that of the NASICON-based materials, but still acceptable when they are combined with conductive carbon.

On the anode side, non-graphitic (hard) carbon can give good results, but only if it is porous and nano-structured. Cobalt compounds have been extensively studied. Among them, cobalt sulfides with ether-based electrolytes are more promising than cobalt oxides for anodes in SIBs, because of their higher electrical conductivity allowing for higher rate capability. However, cobalt is not only toxic, but also very expensive, which constitutes a severe drawback in terms of commercialization. Red or black phosphorous are most attractive anodes. They benefit from the highest capacity, with good cycle ability over a thousand cycles, and they have a good rate capability when they are combined with porous N-doped carbon. Anode materials that are electrochemically active by conversion reaction suffered for a long time from the huge variation of volume during cycling. However, the improvement of their synthesis under the form of nano-structured and porous materials has overcome this problem, since their capacity can now extend to hundreds of cycles. Among them sulfides are of particular interest, since the weak metal-sulfur bonds could kinetically promote the conversion reactions. Moreover, their capacity is enhanced by an important supercapacitance contribution.

Besides electrode shape design and surface modification, the construction of binder-free and self-supporting electrodes has been demonstrated to boost the reaction kinetics and electrode stability. We have given many examples in this review according to which such electrodes are more performing. First of all, they avoid the weight penalty of the binder and additives, so that they allow for a larger loading and energy density. In addition, the polymer binder/conductive additives may cause virtual swelling in common electrolytes, limiting the electrochemical performance, and finally, the absence of binder allows the electrodes to better accommodate the change of volume during cycling. The current challenges of binder-free electrodes and an outlook for their future in energy conversion and storage with focus on advanced SIBs have been detailed in a recent work [391]. Until recently, the construction of binder-free electrodes was not possible with metal active elements because the adhesion of the nano-arrays on the substrate was not maintained due to the huge volume change during cycling associate with the conversion reaction, but a new strategy experienced in the case of Sn proves opens the route to the construction of such anodes. While alloying and doping solve are efficient to improve the structural stability of the electrochemically active materials, the biggest challenge for SIBs is the variation of volume during cycling. A promising strategy to solve this issue is 3D structuring, even in the case of metal-based electrodes, according to the first results published recently.

The constant progress experienced these last five years evidenced in this review gives evidence that the sodium-ion batteries will find an increasing market. They will not compete with the lithium-ion chemistry in terms of energy density and rate capability, and the lithium chemistry will keep the market of the electric cars for instance. However, for static utilization, to buffer the intermittence problem and integration of the production of wind and solar plants to the smart grids, where the volume and weight of the batteries have much less importance than the price and security issues of the batteries, the SIBs should conquest a market.

## Figures and Tables

**Table 1:** Electrochemical properties of selected cathode-materials reviewed in the text, including P2-layered oxide materials (from ref. [43,44,45,46,47,48,49,50,51,52,53,54,55,56,57,58,59,60,61,62,63,64,65,66,67,68,69,70,71,72,73,74,75,76,77,78,79,80,81]), O3-layered oxide materials (from ref. [85,86,87,88,89,90,91,92,93]), another oxide (V_2_O_5_), polyanionic compounds (from ref. [107,108,109,110,111,112,113,114,115,116,117,118,119,120,121,122,123,124,125,126,127,128,129,130,131]), Prussian blue analogs (from ref. [168,169,170,171,172,173,174,175]).

Active Material	Reversible Capacity	Capacity Retention	Ref.
Na_0.7_CoO_2_ spheres (5 μm)	125 mA·h·g^−1^ at 0.04C	86% at 0.4C after 300 cycles	[43]
Na_0.7_CoO_2_ nanosheets	1.16 mA·h·cm^−2^ at 1C	50 mA·h·g^−1^ at 6C after 1100 cycles	[44]
Na_0.67_Ni_0.25_Mg_0.1_Mn_0.65_O_2_	100 mA·h·g^−1^ at C/10	87% at C/10 after 100 cycles	[51]
Na_0.5_[Ni_0.23_Fe_0.13_Mn_0.63_]O_2_	200 mA·h·g^−1^ at 15 mA·h·g^−1^ (C/10)	125 mA·h·g^−1^ at 100 mA·g^−1^ after 100 cycles	[59]
Na_0.85_Li_0.17_Ni_0.21_Mn_0.64_O_2_	95–100 mA·h·g^−1^ at C/10	98% at C/10 after 50 cycles	[65]
Zn-doped Na_0.833_[Li_0.25_Mn_0.75_]O_2_	162 mA·h·g^−1^ at 0.2C	Stable at 0.2C over 200 cycles	[70]
Na_0.7_Mg_0.05_[Mn_0.6_Ni_0.2_Mg_0.15_]O_2_	70 mA·h·g^−1^ at 1C	79% at 1C after 1000 cycles	[73]
Na_0.66_Co_0.5_Mn_0.5_O_2_	86.5 mA·h·g^−1^ at 10C	78.9 mA·h·g^−1^ at 10C over 100 cycles	[77]
Na_2/3_Li_1/9_Ni_5/18_Mn_2/3_O_2_	72.2 mA·h·g^−1^ at 20C	87% at 20C after 1000 cyles	[81]
C-coated NaCrO_2_	120 mA·h·g^−1^ at 20 mA·h·g^−1^ 99 mA·h·g^−1^ at 150C	Stable at 20 mA·g^−1^ over 50 cycles	[85]
Na_0.9_[Cu_0.22_Fe_0.30_Mn_0.48_]O_2_	100 mA·h·g^−1^ electrode at 0.1C	Stable over 100 cycles at 0.1C	[87]
Na[Ni_0.58_Co_0.06_Mn_0.36_]O_2_	157 mA·h·g^−1^ at 15 mA·g^−1^ 132.6 mA·h·g^−1^ at 10C	80% after 300 cycles at 15 mA·g^−1^	[88]
Na_0.75_Ni_0.82_Co_0.12_Mn_0.06_O_2_	80 mA·h·g^−1^ at 1C	65% after 300 cycles at 1C	[90]
NaMn_0.48_Ni_0.2_Fe_0.3_Mg_0.02_O_2_	136 mA·h·g^−1^ at 0.1C	81% after 100 cycles at 0.1C	[93]
V_2_O_5_ nanosheet array	241 mA·h·g^−1^ at 50 mA·g^−1^ 77 mA·h·g^−1^ at 1 A·g^−1^	184 mA·h·g^−1^ after 100 cycles at 100 mA·g^−1^	[104]
Na_3_V_2_(PO_4_)_3_	115 mA·h·g^−1^ at 0.2C 38 mA·h·g^−1^ at 500 C	54% after 1000 cycles at 0.2C	[107]
Na_3_V_2_(PO_4_)_3_/C	86 mA·h·g^−1^ at 100C	64% after 10,000 cycles at 100C	[108]
Na_3_V_2_(PO_4_)_3_/C loading 3.5 mg cm^−2^	96.8 mA·h·g^−1^ at 100C 69.9 mA·h·g^−1^ at 200C	82.0% after 2000 cycles at 20C	[109]
Na_3_V_2_(PO_4_)_3_/C	98.6 mA·h·g^−1^ at 0.5C 78 mA·h·g^−1^ at 192C	91.4% after 2000 cycles at 10C	[110]
Na_3_V_2_(PO_4_)_3_N/C	78.9 mA·h·g^−1^ at 0.1C 59.2 mA·h·g^−1^ at 30C	91.0 % after 800 cycles at 1C 75.9 % after 5000 cycles at 10C	[111]
Na_3_MnZr(PO_4_)_3_	105 mA·h·g^−1^ at 0.1C	91% after 500 cycles at 0.5C	[112]
Na_4_Fe_3_(PO_4_)_2_(P_2_O_7_)	108 mA·h·g^−1^ at 0.1C	69.1% after 4400 cycles at 20C	[115,116]
Na_3_MnTi(PO_4_)_3_/C	160 mA·h·g^−1^ at 0.2C 119 mA·h·g^−1^ at 2C	92 % after 500 cycles at 2C	[113]
Na_3_V_2_(PO_4_)_2_F_3_@C	123 mA·h·g^−1^ at 1C 84 mA·h·g^−1^ at 50C	65% after 5000 cycles at 50C	[122]
Na_3_V_2_(PO_4_)_2_F_3_@C	93.3 mA·h·g^−1^ at 20C	86.3% after 5000 cycles at 20C	[127]
Na_3_(VOPO_4_)_2_F	112 mA·h·g^−1^ at C/5 100 mA·h·g^−1^ at 2C	93.8 % after 200 cycles at C/5 90% after 1200 cycles at 2C	[129]
Na_3_(VOPO_4_)_2_F load 2.0 mg cm^−2^	125 mA·h·g^−1^ at 1C	88% after 1000 cycles at 5C	[131]
Na_2+2x_Fe_2-x_(SO_4_)_3_@rGO	99 mA·h·g^−1^ at 0.1C 78 mA·h·g^−1^ at 60C	80.8% after 2000 cycles at 30C	[143]
Na_2.3_Cu_1.1_Mn_2_O_7-d_	106.6 mA·h·g^−1^ at 20C	95.8% after 1000 cycles at 20C	[151]
Na_2_FeP_2_O_7_@rGO	128 mA·h·g^−1^ at 0.1C 35.1 mA·h·g^−1^ at 200C	62.3% after 6000 cycles at 10C	[156]
Na_0.81_Fe[Fe(CN)_6_]_0.79–0.61_@rGO	163 mA·h·g^−1^ at 30 mA·g^−1^ 112 mA·h·g^−1^ at 800 mA·g^−1^	91.9% after 500 cycles at 200 mA·g^−1^	[168]
Na_2_CoFe(CN)_6_	150 mA·h·g^−1^ at 0.1C	90% after 200 cycles at 0.1C	[173]
Ni_0.67_Fe_0.33_Se_2_	450 mA·h·g^−1^ at 10 A·g^−1^	375 mA·h·g^−1^ after 10,000 cycles at 10 A·g^−1^	[175]

**Table 2:** Electrochemical properties of selected anode materials reviewed in the text: carbon-based anodes (from ref. [187,188,189,190,191,192,193,194,195,196,197,198,199,200,201,202,203,204,205,206,207,208,209,210,211,212,213,214,215,216,217,218,219]), metal chalcogenide-based including intercalation materials (from ref. [231,232,233,234,235,236,237,238,239,240,241,242,243,244,245]) and conversion reaction compounds (from ref. [259]).

Active Material	Reversible Capacity	Capacity Retention	Ref.
3D porous graphene + Al_2_O_3_	140 mA·h·g^−1^ at 0.5 A·g^−1^	82.9% after 500 cycles at 0.5 A·g^−1^	[187]
Hollow carbon nanowires	251 mA·h·g^−1^ at 0.2C 149 mA·h·g^−1^ at 2C	200 mA·h·g^−1^ after 200 cycles at 125 mA·g^−1^	[193]
Highly disordered carbon	225 mA·h·g^−1^ at 100 mA·g^−1^	92 % after 180 cycles at 100 mA·g^−1^	[194]
Carbon nano-fibers	173 mA·h·g^−1^ at 0.2 A·g^−1^ 82 mA·h·g^−1^ at 2 A·g^−1^	97.70% after 200 cycles at 0.2 A·g^−1^	[195]
Hard-carbon	112 mA·h·g^−1^ at 5C	85 mA·h·g^−1^ after 1000 cycles at 5C	[198]
N-rich mesoporous carbon	338 mA·h·g^−1^ at 30 mA·g^−1^	111 mA·h·g^−1^ 800 cycles at 500 mA·g^−1^	[202]
N-doped carbon sheets	292 mA·h·g^−1^ at 0.15C	50 mA·h·g^−1^ over 2000 cycles at 4.5C	[203]
N/S co-doped mesoporous carbon	419 mA·h·g^−1^ at 0.1 A·g^−1^	419 mA·h·g^−1^ at 150 cycles at 0.1 A·g^−1^; 220 mA·h·g^−1^ at 3000 cycles at 5 A·g^−1^	[205]
S-doped carbon nanosheets	605 mA·h·g^−1^ at 50 mA·g^−1^ 133 mA·h·g^−1^ at 10 A·g^−1^	211 mA·h·g^−1^ upon 2000 cycles at 5 A·g^−1^	[207]
Carbon quantum dots	150 mA·h·g^−1^ at 2.5 A·g^−1^	150 mA·h·g^−1^ over 3000 cycles at 2.5 A·g^−1^; 100 mA·h·g^−1^ 10,000 cycles at 5 A·g^−1^	[219]
P-TiO_2_ nanotube arrays	334 mA·h·g^−1^ at 67 mA·g^−1^	141 mA·h·g^−1^ after 1000 cycles at 3.35 A·g^−1^	[231]
TiO2 nanorods	155 mA·h·g^−1^ at 5 A·g^−1^	149 mA·h·g^−1^ over 2000 cycles at 1A·g^−1^	[233]
Graphene-TiO_2_	115 mA·h·g^−1^ at 1 A·g^−1^	102 mA·h·g^−1^ after 300 cycles at 0.1 A·g^−1^	[237]
Graphene-TiO_2_	120 mA·h·g^−1^ at 2C	Stable over 4300 cycles at 2C	[238]
C-coated TiO_2_	1227 mA·h·g^−1^ at 0.1C 134 mA·h·g^−1^ at 10C	Full retention up to 300th cycle at 1C	[239]
C-coated TiO_2_ hollow sphere	204.8 mA·h·g^−1^ at 0.5C	140 mA·h·g^−1^ after 500 cycles at 5C	[240]
TiO_2_ + C-dots	264 mA·h·g^−1^ at 0.1C	108.2 mA·h·g^−1^ after 2000 cycles at 10C	[241]
N-doped TiO_2_ nanorods + C-dots	185 mA·h·g^−1^ at 10C	91.6% after 1000 cycles at 10C	[242]
Graphene + TiO_2_ films	-	72 mA·h·g^−1^ at 10,000th cycle at 5 A·g^−1^	[245]
Fe_3_O_4_@N-doped carbon	781 mA·h·g^−1^ at 1 A·g^−1^	522 mA·h·g^−1^ after 800th cycle at 160 mA·g^−1^	[259]
C/Fe_2_O_3_	317 mA·h·g^−1^ at 8 A·g^−1^	740 mA·h·g^−1^ after 200 cycles	[261]
Fe_2_O_3_ embedded in N-doped C	155.3 mA·h·g^−1^ at 4 A·g^−1^	474 mA·h·g^−1^ at 100th cycle at 100 mA·g^−1^	[262]
Co_3_O_4_	204 mA·h·g^−1^ at 445 mA·g^−1^	80% after 200 cycles at 445 mA·g^−1^	[266]
Co_3_O_4_	707 mA·h·g^−1^ at 90 mA·g^−1^	416 mA·h·g^−1^ at 100th cycle at 90 mA·g^−1^	[267]
Co_3_O_4_/N-doped graphite	145 mA·h·g^−1^ at 2 A·g^−1^	214 mA·h·g^−1^ over 100 cycles at 0.1 A·g^−1^; 120 mA·h·g^−1^ over 2000 cycles at 0.5 A·g^−1^	[271]
C-confined Co_3_O_4_	712 mA·h·g^−1^ at 0.1 A·g^−1^ 223 mA·h·g^−1^ at 5 A·g^−1^	74.5% after 500 cycles	[280]
Graphene/SnO_2_/Co_3_O_4_	-	461 mA·h·g^−1^ after 80 cycles at 0.1 A·g^−1^; 241 mA·h·g^−1^ after 500 cycles at 1 A·g^−1^	[282]
CuO rod arrays	640 mA·h·g^−1^ at 200 mA·g^−1^	290 mA·h·g^−1^ at 450th cycle at 200 mA·g^−1^	[285]
CuO + 44 wt.% C	304 mA·h·g^−1^ at 2 A·g^−1^	402 mA·h·g^−1^ at 600th cycle at 200 mA·g^−1^	[287]
3D porous carbon encapsulated SnO_2_	100 mA·h·g^−1^ at 1.6 A·g^−1^	280 mA·h·g^−1^ at 250th cycle at 100 mA·g^−1^	[288]
SnO_2_/C	150 mA·h·g^−1^ at 1 A·h·g^−1^	293 mA·h·g^−1^ at 100th cycle at 50 mA·g^−1^	[289]
a-SnO_2_ ordered arrays	200 mA·h·g^−1^ at 2 A·g^−1^	220 mA·h·g^−1^ after 800 cycles at 1 A·g^−1^	[290]
Co_3_S_4_@N-rich C	284 mA·h·g^−1^ at 1 A·g^−1^	421 mA·h·g^−1^ at 100th cycle at 100 mA·g^−1^	[293]
NiCo_2_S_4_ nanosheets	-	387 mA·h·g⁻^1^ after 60 cycles at 1 A·g^−1^	[294]
rGO + NiCo_2_S_4_	220 mA·h·g^−1^ at 800 mA·g^−1^	530 mA·h·g^−1^ after 70 cycles at 50 mA·g^−1^	[295]
NiCo_2_S_4_ nanodots/N-doped carbon	530 mA·h·g^−1^ at 1 A·g^−1^	570 mA·h·g^−1^ over 200 cycles at 0.2 A·g^−1^; 395 mA·h·g^−1^ at 6 A·g^−1^ after 5000 cycles	[296]
Co_9_S_8_ quantum dots + C	472 mA·h·g^−1^ at 0.1 A·g^−1^	340 mA·h·g^−1^ after 2000 cycles at 1 A·g^−1^	[299]
Co_9_S_8_ quantum dot/hollow carbon matrix/graphene	330 mA·h·g^−1^ at 6.4 A·g^−1^	628 mA·h·g^−1^ at 500th cycle at 300 mA·g^−1^	[301]
Co_9_S_8_/MoS_2_ + N-doped C	438 mA·h·g^−1^ at 1.0 A·g^−1^	421 mA·h·g^−1^ after 250 cycles at 2.0 A·g^−1^	[302]
MoS_2_@ carbon nanofiber interpenetrated graphene	598 mA·h·g^−1^ at 0.1 A·g^−1^	412 mA·h·g^−1^ at 1000th cycle at 1 A·g^−1^ 366 mA·h·g^−1^ at 1000th cycle at 5 A·g^−1^	[305]
3D carbon networks/Fe_7_S_8_/graphene	2.12 mA·h cm^−2^ at 0.25 mA·cm^−2^	47.5% loss after 5000 cycles at 0.25 mA·cm^−2^	[330]
SnS nanoflakes/doped C	250.7 mA·h·g^−1^ at 20 A·g^−1^	∼98% after 100 cycles at 1 A·g^−1^	[333]
SnS/C nanofibers	786 mA·h·g^−1^ at 100 mA·g^−1^	481 mA·h·g^−1^ after 100 cycles at 50 mA·g^−1^ 349 mA·h·g^−1^ after 500 cycles at 200 mA·g^−1^	[334]
ZnS-SnS@C	916 mA·h·g^−1^ at 200 mA·g^−1^	302 mA·h·g^−1^ after 500 cycles at 500 mA·g^−1^	[335]
SnS_2_ in N- and S-doped C	310 mA·h·g^−1^ at 4 A·g^−1^	380 mA·h·g^−1^ after 200 cycles at 500 mA·g^−1^	[336]
SnS_2_/Mn_2_SnS_4_/carbon	488 mA·h·g^−1^ at 10 A·g^−1^	522 mA·h·g^−1^ after 500 cycles at 5 A·g^−1^	[337]
SnS_2_ nanosheet arrays	647 mA·h·g^−1^ at 50 mA·g^−1^	631 mA·h·g^−1^ at 150th cycle at 50 mA·g^−1^	[339]
NiS_2_	200 mA·h·g^−1^ at 2.0 A·g^−1^	120 mA·h·g^−1^ at 2000th cycle at 2.0 A·g^−1^	[343]
Polypyrrole coated CoP wires	0.285 mA·h·cm^−2^ at 3 mA·cm^−2^	0.443 mA·h cm^−2^ at 1.5 mA·cm^−2^ stable over 1000 cycles	[349]
CoP@C-RGO-Ni foam	543 mA·h·g^−1^ at 200 mA·g^−1^	473 mA·h·g^−1^ after 100 cycles at 100 mA·g^−1^	[350]
Sn-Cu	420 mA·h·g^−1^ at 0.2C	97% after 100 cycles at 0.2C	[356]
Sn particles on N-doped graphite	429 mA·h (g_Sn+C_)^−1^ at 0.2C	290 mA·h (g_Sn+C_)^−1^ after 1000 cycles at 1 A·g^−1^	[357]
Sn nano array on Cu	801 mA·h·g^−1^ at 0.2 C 610 mA·h·g^−1^ at 5 C	501 mA·h·g^−1^ after 300 cycles at 5 C	[356]
Sb on C nanofibers	631 mA·h·g^−1^ at C/15	90% after 400 cycles at C/3 (200 mA·g^−1^)	[360]
Sb/N-doped porous C	529 mA·h·g^−1^ at 100 mA·g^−1^	97% after 100 cycles at 100 mA·g^−1^	[363]
Sb/N-doped C	440 mA·h·g^−1^ at 100 mA·g^−1^	328 mA·h·g^−1^ after 300 cycles at 100 mA·g^−1^	[364]
